# Accelerated free‐breathing whole‐heart 3D T_2_ mapping with high isotropic resolution

**DOI:** 10.1002/mrm.27989

**Published:** 2019-09-19

**Authors:** Aurélien Bustin, Giorgia Milotta, Tevfik F. Ismail, Radhouene Neji, René M. Botnar, Claudia Prieto

**Affiliations:** ^1^ Department of Biomedical Engineering School of Biomedical Engineering and Imaging Sciences King's College London London United Kingdom; ^2^ MR Research Collaborations, Siemens Healthcare Frimley United Kingdom; ^3^ Escuela de Ingeniería Pontificia Universidad Católica de Chile Santiago Chile

**Keywords:** fast imaging, isotropic resolution, motion correction, myocardial T_2_ mapping, T_2_ quantification

## Abstract

**Purpose:**

To enable free‐breathing whole‐heart 3D T_2_ mapping with high isotropic resolution in a clinically feasible and predictable scan time. This 3D motion‐corrected undersampled signal matched (MUST) T_2_ map is achieved by combining an undersampled motion‐compensated T_2_‐prepared Cartesian acquisition with a high‐order patch‐based reconstruction.

**Methods:**

The 3D MUST‐T_2_ mapping acquisition consists of an electrocardiogram‐triggered, T_2_‐prepared, balanced SSFP sequence with nonselective saturation pulses. Three undersampled T_2_‐weighted volumes are acquired using a 3D Cartesian variable‐density sampling with increasing T_2_ preparation times. A 2D image‐based navigator is used to correct for respiratory motion of the heart and allow 100% scan efficiency. Multicontrast high‐dimensionality undersampled patch‐based reconstruction is used in concert with dictionary matching to generate 3D T_2_ maps. The proposed framework was evaluated in simulations, phantom experiments, and in vivo (10 healthy subjects, 2 patients) with 1.5‐mm^3^ isotropic resolution. Three‐dimensional MUST‐T_2_ was compared against standard multi‐echo spin‐echo sequence (phantom) and conventional breath‐held single‐shot 2D SSFP T_2_ mapping (in vivo).

**Results:**

Three‐dimensional MUST‐T_2_ showed high accuracy in phantom experiments (R^2^ > 0.99). The precision of T_2_ values was similar for 3D MUST‐T_2_ and 2D balanced SSFP T_2_ mapping in vivo (5 ± 1 ms versus 4 ± 2 ms, *P* = .52). Slightly longer T_2_ values were observed with 3D MUST‐T_2_ in comparison to 2D balanced SSFP T_2_ mapping (50.7 ± 2 ms versus 48.2 ± 1 ms, *P* < .05). Preliminary results in patients demonstrated T_2_ values in agreement with literature values.

**Conclusion:**

The proposed approach enables free‐breathing whole‐heart 3D T_2_ mapping with high isotropic resolution in about 8 minutes, achieving accurate and precise T_2_ quantification of myocardial tissue in a clinically feasible scan time.

## 1 INTRODUCTION

Quantitative myocardial T_2_ mapping has emerged as a promising tool for edema characterization and detection of subtle myocardial inflammation in patients with acute myocardial infarction, myocarditis, dilated cardiomyopathy, sarcoidosis, and autoimmune cardiomyopathies.^1^, ^2^, ^3^, ^4^

Myocardial T_2_ mapping is usually performed by acquiring several electrocardiogram (ECG)‐triggered T_2_‐weighted images, with different amounts of T_2_ decay through T_2_‐preparation pulses. A map of T_2_ relaxation times is then generated by fitting the series of weighted images to an exponential decay model on a pixel‐by‐pixel basis. Current clinical protocols usually perform myocardial T_2_ mapping with a 2D single‐shot SSFP (T_2_p‐SSFP), acquiring 3 T_2_‐prepared images in a single breath‐hold every 2 to 3 heartbeats to allow for full T_1_ recovery.^3^ Multiple short‐axis slices are usually acquired at the basal, midventricular, and apical level.

However, the use of 2D acquisitions with fairly thick slices and the associated partial volume effects undermine the full potential of myocardial T_2_ mapping, particularly in hypertrophic cardiomyopathies in which the pathological tissues are often complex 3D structures with differing T_2_ values.^5^, ^6^, ^7^ Furthermore, 2D sequences, typically acquired during breath‐holding, regularly suffer from respiratory and cardiac motion between the T_2_‐weighted images, due primarily to imperfect breath‐holding or variable heart rate.^8^ Although robust nonrigid motion‐correction techniques have been proposed to correct for residual motion and improve map quality, such techniques are relatively complex, computationally expensive, and only correct for in‐plane motion.^9^, ^10^, ^11^

A free‐breathing 3D T_2_ mapping approach with high isotropic spatial resolution may have the potential to increase diagnostic accuracy in patients with acute myocardial injury (e.g., NSTEMI patients^12^, ^13^) and improve the detection of cardiac involvement in patients with systemic sarcoidosis.^14^

Three‐dimensional myocardial T_2_ mapping techniques have been proposed to address the limitations of 2D imaging by allowing for large coverage of the heart with inherently higher SNR. Non‐Cartesian self‐navigated myocardial T_2_ mapping has been proposed to perform free‐breathing 3D myocardial T_2_ mapping at 3 T in about 18‐minute acquisition time.^5^, ^15^ Such long scan times, unfortunately, may impede the clinical integration of this technique. A self‐navigated hybrid radial Cartesian trajectory was also proposed to perform free‐breathing 3D myocardial T_2_ mapping in less than 5 minutes, although with low spatial resolution.^16^ The use of saturation pulses at every heartbeat was proposed to accelerate Cartesian 3D T_2_ mapping.^17^ Long resting periods (in the order of 2‐3 heartbeats^3^, ^5^) are usually needed in T_2_ mapping to ensure full recovery of the longitudinal magnetization between acquisitions to minimize T_1_ effects and satisfy the assumption that the equilibrium state magnetization is not affected by heart‐rate variations throughout the scan. Nonselective 90° saturation pulses reset magnetization history by tipping the longitudinal magnetization of the imaged volume into the transverse plane, thus removing heart‐rate dependency during the acquisition and allowing for imaging every heartbeat. This approach also eliminates artifacts caused by heart‐rate variations, at the cost of a reduced SNR and lower precision of T_2_ values. However, this technique uses prospective diaphragmatic navigator‐gated acquisitions, thus leading to prolonged and often unpredictable scan times (about 9‐minute nominal scan time with 30% to 60% respiratory gating efficiency), as only a small fraction of the acquired data is accepted for reconstruction (referred to as low scan efficiency).

Notwithstanding the continued advancements, myocardial 3D T_2_ mapping still faces substantial technical challenges that in turn may limit its applicability in clinical practice. In this study, we sought to achieve high isotropic spatial resolution 3D Cartesian whole‐heart myocardial T_2_ mapping in a short and predictable scan time of about 8 minutes by combining a highly accelerated saturation‐based free‐breathing myocardial T_2_ mapping sequence with 2D image‐based navigator (iNAV) respiratory motion correction. A recently proposed high‐dimensionality undersampled patch‐based reconstruction (HD‐PROST)^18^ is used in concert with dictionary matching, based on the extended phase graph formalism,^19^ to allow for accurate and precise myocardial T_2_ maps. The proposed framework was validated in simulations, phantom, and healthy subject experiments, while its initial clinical feasibility was shown for 2 patients with suspected cardiovascular disease.

## 2 METHODS

Acquisitions were performed on a 1.5T MR scanner (Magnetom Aera, Siemens Healthcare, Erlangen, Germany) with a dedicated 18‐channel body coil and a 32‐channel spine coil. Written informed consent was obtained from all healthy subjects and patients before undergoing MRI scans, and the study was approved by the National Research Ethics Service. Numerical simulations, reconstructions and analysis were performed on a workstation with a 16‐core dual Intel Xeon Processor (2.3 GHz, 256 GB RAM).

### 2.1 Accelerated whole‐heart 3D T_2_ mapping sequence

**Figure 1:**
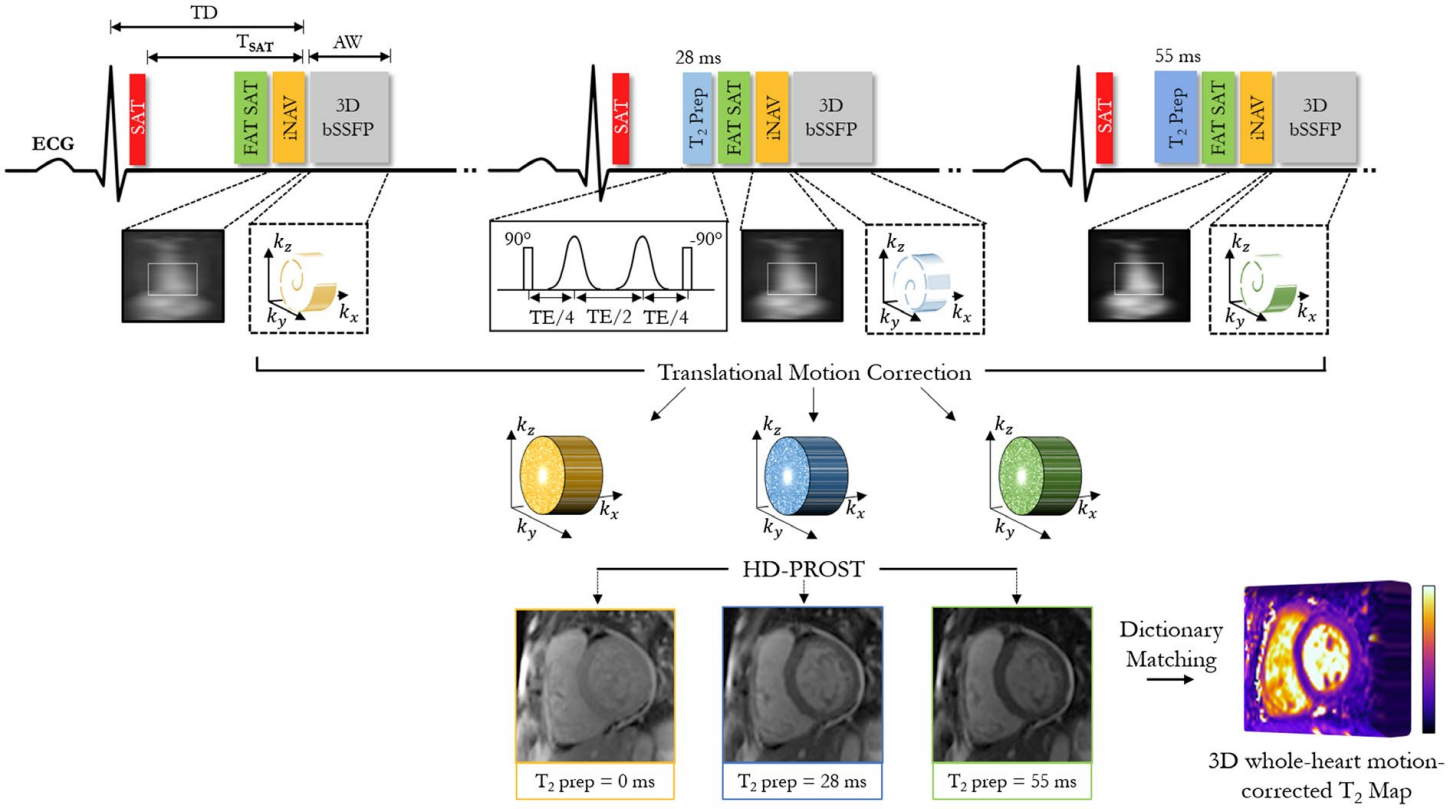
Schematic overview of the proposed free‐breathing 3D motion‐corrected undersampled signal matched (MUST) T_2_ technique for whole‐heart myocardial T_2_ mapping. Three T_2_‐prepared volumes are acquired sequentially with increasing TE_T2prep_ ([0,28,55] ms). A nonselective saturation pulse is applied immediately after the electrocardiogram (ECG) R‐wave to avoid recovery heartbeats. A 2D image navigator is acquired to enable translational respiratory motion correction of the heart and shorter and predictable scan times. A golden‐angle shifted variable‐density Cartesian undersampling is used to achieve clinically feasible scan times. All T_2_ prepared volumes are reconstructed simultaneously with high‐dimensionality undersampled patch‐based reconstruction (HD‐PROST).^18^ A dictionary is then simulated and matched to the measured signal to generate the whole‐heart T_2_ maps. Abbreviations: AW, acquisition window; bSSFP, balanced SSFP; iNAV, image‐based navigator; TD, trigger delay; T_SAT_, saturation time

The proposed myocardial 3D motion‐corrected undersampled signal‐matched (MUST) T_2_ mapping sequence is shown in Figure 1. A saturation pulse is performed right after the R‐wave with a constant saturation time (T_SAT_) from the data acquisition. The value of T_SAT_ is set to the maximal available time allowed by the sequence to guarantee enough magnetization recovery before the next acquisition. This approach allows efficient imaging at every heartbeat, avoiding the use of multiple recovery periods.^17^ Subsequently, an adiabatic T_2_ prepared pulse with variable TE (TE_T2prep_) is performed, followed by a balanced SSFP readout. An adiabatic T_2_‐prep module (Silver‐Hoult type) is used because of its good insensitivity to B_1_ and B_0_ inhomogeneities.^20^ Three T_2_‐weighted volumes are acquired sequentially with different T_2_ weightings (TE_T2prep_ = [0, 28, 55] ms).

A 3D Cartesian variable‐density trajectory with spiral profile order^21^, ^22^ is used to allow for fast acquisition of the 3 T_2_‐prepared volumes. This trajectory samples the k_y_‐k_z_ phase‐encoding plane following approximate spiral interleaves on the Cartesian grid with variable density along each spiral arm and with 2 successive spiral interleaves being rotated by the golden angle. A golden angle rotation^21^, ^23^ between different contrast acquisitions was incorporated to introduce incoherently distributed aliasing along the contrast dimension and noise‐like artifacts in the spatial dimension (Supporting Information Figure S1).

A 2D low‐resolution iNAV^24^ precedes the 3D acquisition at every heartbeat to enable 2D translational respiratory motion estimation/compensation (superior–inferior and right–left directions). This technique, already validated for coronary MRA^24^ and multicontrast cardiac MRI,^25^ enables 100% scan efficiency with predictable scan time, resulting in about 2‐3 times acceleration compared with diaphragmatic navigator gate–based acquisitions. Two‐dimensional iNAVs are obtained by spatially encoding the startup echoes of the balanced SSFP T_2_‐mapping sequence. Two‐dimensional translational motion is estimated using a template‐matching algorithm with normalized cross correlation as similarity measure, with the template manually selected around the heart during acquisition planning. Motion compensation of the 3 volumes is performed by modulating the k‐space data with a linear phase shift to a reference respiratory position selected at end‐expiration.^24^

### 2.2 Multicontrast HD‐PROST reconstruction

Multicontrast HD‐PROST^18^, ^22^ is used to reconstruct the 3 undersampled T_2_‐weighted volumes. The HD‐PROST reconstruction exploits local (i.e., within a patch), nonlocal (i.e., between similar patches within a neighborhood), and contrast (i.e., between T_2_‐weighted images) redundancies of the 3D volumes in an efficient low‐rank formulation. The reconstruction is formulated as an iterative 2‐step process: (1) an L2‐norm regularized parallel‐imaging reconstruction using the denoised multicontrast data from step 2 as a prior (optimization 1); and (2) an efficient high‐order low‐rank patch‐based denoising (optimization 2, Supporting Information Figure S2). The first step is optimized by gradient descent, whereas the second step is optimized through high‐order low‐rank tensor decomposition. The performance of HD‐PROST reconstruction relies primarily on 2 parameters that need to be carefully tuned to get the best reconstruction quality: (1) the patch size (*N* = 5 × 5 × 5 pixels in this study), reflecting the degree of structural information within each patch, and (2) the high‐order singular value truncation parameter (*λ_p_* = 0.1 in this study) that controls the amount of regularization.

The HD‐PROST reconstruction was implemented and performed offline using the algorithm described in Bustin et al.^18^ Reconstruction parameters were optimized empirically (as previously reported) and are illustrated in Supporting Information Figure S2. We refer the reader to Bustin et al^18^, ^26^ for a detailed description of HD‐PROST and the associated reconstruction parameters.

### 2.3 Numerical simulations

Usually in myocardial T_2_ mapping, the acquisition of each T_2_‐weighted image is followed by a resting period of about 2‐3 heartbeats to allow for full longitudinal magnetization relaxation. Numerical extended phase graph simulations were first performed to investigate the impact of removing recovery heartbeats and integrating saturation pulses on the longitudinal magnetization of 3D MUST‐T_2_ for tissues with different relaxation times T_1_. Simulations were performed for a T_2_ of 50 ms and varying T_1_ values (ranging from 700 ms to 1200 ms) with and without saturation pulses. Other relevant parameters used in the simulations were TE_T2prep_ = 55 ms, TR = 3.2 ms, trigger delay = 856 ms, data‐acquisition window of 100 ms, and a simulated heart rate of 60 beats per minute (bpm).

### 2.4 Dictionary generation and matching

Three‐dimensional MUST‐T_2_ dictionaries are simulated using the extended phase graph formalism^19^, ^27^, ^28^ for a range of T_2_ values. For both phantom and in vivo experiments, the dictionaries are calculated matching the subject‐specific acquisition parameters with T_2_s in the range [minimum : step size : maximum] of [4:2:100, 105:5:200, 210:10:450] ms. Simulations show that the proposed method is not dependent on the used T_1_ value (in the range of interest) for dictionary generation (Supporting Information Figure S3, where the simulation results are shown for different T_1_ values ranging from 850 ms to 1200 ms with a step size of 50 ms); thus, T_1_ is kept constant at 1100 ms for the phantom and in vivo experiments. The T_2_ maps are obtained by voxel‐wise matching of the measured and normalized signal to the closest (minimum least squares) extended phase graph–based dictionary entry. The choice of dictionary‐based matching versus conventional mono‐exponential curve fitting is justified in Supporting Information Figure S4.

### 2.5 Phantom experiments

The proposed ECG‐triggered 3D MUST‐T_2_ mapping sequence was evaluated in a standardized (T1MES) T_1_/T_2_ phantom containing 9 agarose‐based tubes with relevant cardiac T_1_ and T_2_ combinations (range, T_1_: 255‐1489 ms, T_2_: 44‐243 ms).^29^ Scan parameters for 3D MUST‐T_2_ included TR = 3.13 ms, TE = 1.37 ms, flip angle = 90°, FOV = 187 × 187 × 156 mm^3^, 1.5 mm^3^ isotropic resolution, bandwidth = 908 Hz/pixel, data‐acquisition window = 100 ms, and TE_T2prep_ = [0, 28, 55] ms. Reference T_2_ relaxation times for each vial were obtained using a multi‐echo spin‐echo (SE) sequence with 8 TEs ranging from 10 ms to 640 ms. In addition, conventional ECG‐triggered 2D T_2_p‐SSFP was performed for comparison purposes with the following imaging parameters: 1.9 × 1.9 mm^2^ in‐plane resolution, slice thickness = 8 mm, TR/TE = 3.11/1.38 ms, linear k‐space reordering, bandwidth = 1184 Hz/pixel, TE_T2prep_ = [0, 25, 55] ms, flip angle = 70°, 3 recovery beats, and GRAPPA factor 2 with 36 k‐space autocalibration lines. The T_2_ relaxation times were obtained by mono‐exponential curve fitting for both the SE and T_2_p‐SSFP sequences.

#### 2.5.1 Impact of heart rate on T_2_ accuracy

The impact of heart‐rate differences on T_2_ accuracy was assessed by performing multiple 3D MUST‐T_2_ phantom acquisitions with different simulated heart rates (ranging from 50 bpm to 100 bpm, step size 10 bpm). The trigger delay was set to 70% of the R‐R interval and ranged from 420 ms to 840 ms, while the saturation time T_SAT_ was always set to the maximum allowed time (ranging from 370 ms to 790 ms). The acquisitions were performed fully sampled using the previously described variable‐density trajectory with spiral profile order sampling.

#### 2.5.2 Impact of acceleration on T_2_ accuracy

To study the impact of accelerating 3D MUST‐T_2_ on the accuracy of the T_2_ values, several phantom acquisitions were performed with different undersampling factors ranging from 1‐fold to 5‐fold (step size 1). Additional acquisition parameters included simulated heart rate of 60 bpm, trigger delay of 680 ms, and saturation time T_SAT_ = 630 ms.

#### 2.5.3 Data analysis

Circular regions of interest were drawn on each vial for 3D MUST‐T_2_, SE, and 2D T_2_p‐SSFP sequences. For each vial, the average T_2_ value was measured, and agreement among SE, 2D T_2_p‐SSFP T_2_ values, and the proposed approach was assessed using linear regression.

### 2.6 In vivo experiments

#### 2.6.1 Healthy subjects

Ten healthy subjects (5 men and 5 women, mean age of 30 years [range: 26‐36 years]) with no history of cardiovascular disease underwent ECG‐triggered free‐breathing 3D whole‐heart T_2_ mapping using the proposed 3D MUST‐T_2_ acquisition approach. Relevant scan parameters included FOV = 320 × 320 × 84‐108 mm^3^, slice oversampling = 22%, 1.5‐mm^3^ isotropic resolution, flip angle = 90°, TE_T2prep_ = [0, 28, 55] ms, subject‐dependent middiastolic trigger delay (mean: 672 ms [range: 521‐952 ms]), acquisition window (mean: 97 ms [range: 80‐108 ms]), T_SAT_ (range: 470‐900 ms), acceleration factor of ×5, 14 linear ramp‐up pulses for iNAV, and Hanning‐filtered sinc pulse with a duration of 1 ms and time‐bandwidth product of 4.5. To ensure adequate fat suppression, a spectral presaturation with inversion recovery was applied before imaging. All 3D MUST‐T_2_ mapping acquisitions were performed in the coronal plane.

Conventional 2D T_2_p‐SSFP T_2_ mapping^3^ was acquired for each subject for comparison purposes. Acquisition parameters were the same as for the phantom experiments. Three short‐axis slices (basal, midcavity, apical) were acquired in 3 separate breath‐holds of about 10 seconds each. A nonrigid motion correction was carried out inline to compensate for in‐plane motion between the three 2D T_2_‐weighted images.^11^ Subsequently, T_2_ maps were obtained by mono‐exponential curve fitting.

#### 2.6.2 Patients

The feasibility and preliminary clinical performance of the proposed 3D MUST‐T_2_ sequence was assessed in 2 patients (2 men, ages 42 and 50 years) with suspected cardiovascular disease. The same acquisition and reconstruction parameters as in the healthy subject study were used. The patient‐specific saturation times were T_SAT_ = 525 ms (patient 1, average heart rate = 75 bpm, acquisition window = 98 ms) and T_SAT_ = 595 ms (patient 2, average heart rate = 65 bpm, acquisition window = 100 ms). Conventional 2D T_2_p‐SSFP T_2_ mapping was acquired for each patient with the same parameters as in the healthy subject study.

#### 2.6.3 Data analysis

The 3D MUST‐T_2_ maps were reformatted in the short‐axis view to match the 2D T_2_p‐SSFP slices. Regional differences in left ventricular T_2_ relaxation times were assessed according to the 16‐segment model of the American Heart Association.^30^ The T_2_ measurements were reported as mean ± SD and assessed using the Student's t‐test. Statistical differences in regional T_2_ values among reformatted basal, midventricular, and apical short‐axis slices were analyzed using repeated‐measures 1‐way analysis of variance with Bonferroni post hoc comparisons. All statistical analyses were performed using MATLAB (version 7.1, MathWorks, Natick, MA), and statistical significances were defined as a *P*‐value < .05.

## 3 RESULTS

### 3.1 Numerical simulations

**Figure 2:**
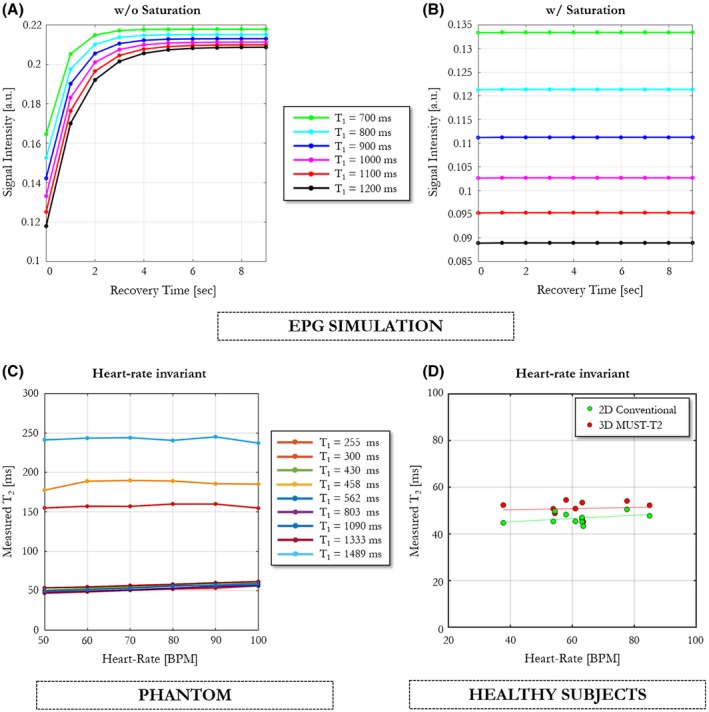
Results from extended phase graph (EPG) simulations show the effect of the saturation pulse on the MR signal evolution. A, Simulated magnetization obtained with the EPG formalism for different recovery times (ranging from 0 to 9 seconds) when the saturation pulse is not used. The signals were generated for tissues with a T_2_ of 50 ms, varying T_1_s (ranging from 700 ms to 1200 ms), TE_T2prep_ = 50 ms, and a simulated heart rate of 60 bpm. For long T_1_s, a minimum of about 6 idle heartbeats are needed to allow for full recovery of the longitudinal magnetization. B, When the saturation pulse is applied at every heartbeat, idle heartbeats are not required for signal recovery, at the cost of lower signal intensity. C, Evolution of the matched T_2_ values obtained with the proposed 3D MUST‐T_2_ mapping sequence over different simulated heart rates (ranging from 50 bpm to 100 bpm) for each phantom vial. The proposed approach is mostly insensitive to heart‐rate variations, even for long T_1_s. D, Effect of different heart rates across all healthy subjects (N = 10) on mean T_2_ values. Abbreviations: BPM, beats per minute

The impact of recovery times on the signal intensity (longitudinal magnetization) is depicted in Figure 2A,B for multiple T_1_ relaxation times (ranging from 700 ms to 1200 ms). Although a clear dependency was observed when no saturation pulse was applied, necessitating about 2‐3 resting periods to recover 96% of the signal intensity (Figure 2A), applying a “reset” saturation pulse^17^ at every heartbeat avoids recovery periods, at the cost of a lower signal intensity (Figure 2B).

### 3.2 Phantom experiments

The average T_2_ values obtained in the phantom experiments when increasing the simulated heart rate (from 50 bpm to 100 bpm) are depicted in Figure 2C. The T_2_ relaxation times measured with the proposed 3D MUST‐T_2_ sequence showed no to little sensitivity to change of heart rate for all vials in comparison to SE reference measurements.

**Figure 3:**
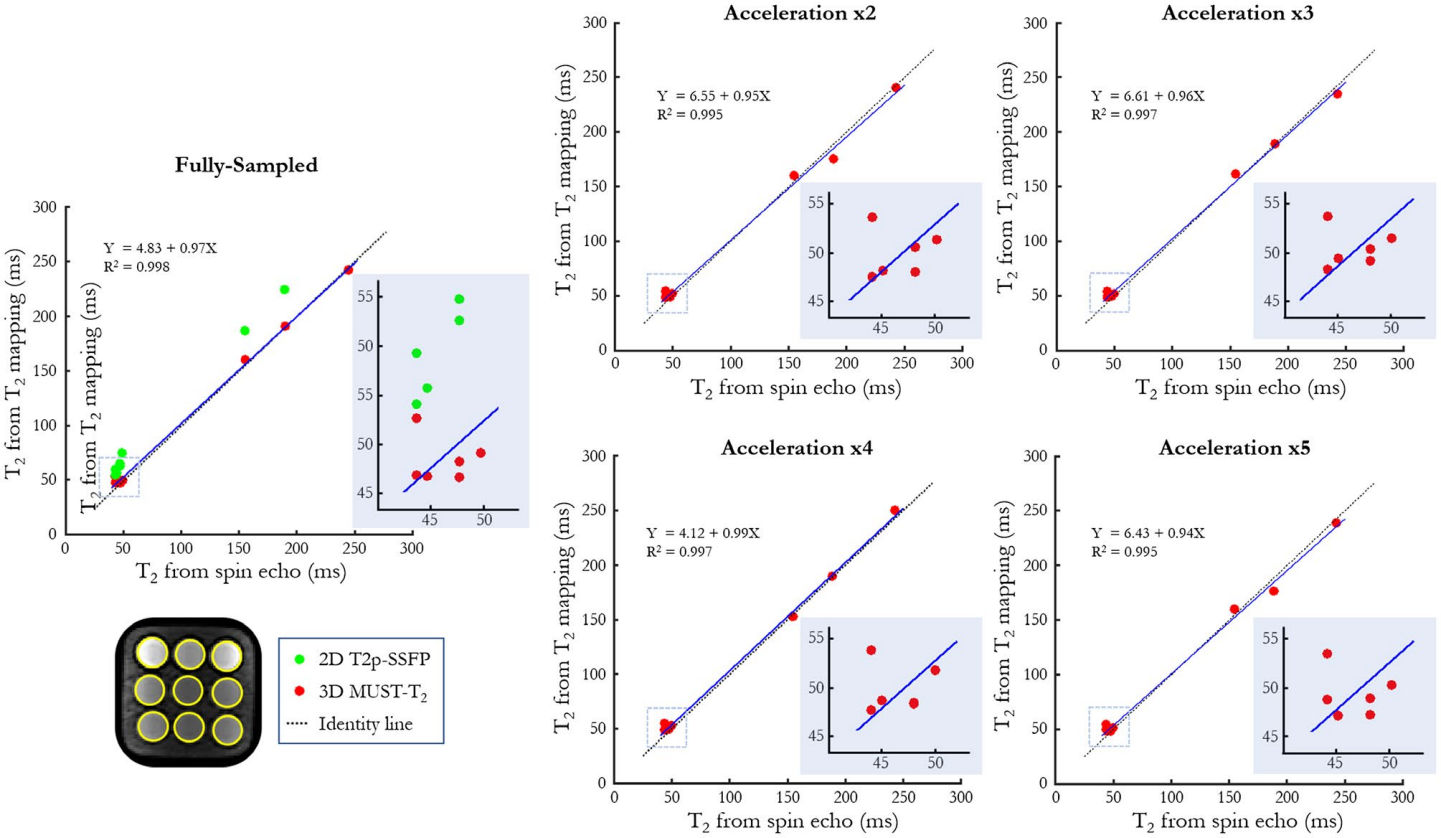
Phantom accuracy for the proposed 3D MUST‐T_2_ sequence. Plots compare the mean T_2_ values derived from the 9 vials for 5 different acceleration factors with the ground‐truth T_2_ values (measured by spin echo [SE] with 8 TEs from 10‐640 ms^29^), conventional 2D T_2_p‐SSFP mapping (green), and the proposed 3D MUST‐T_2_ sequence. The T_2_ accuracy is preserved with the proposed approach with excellent agreement with the reference T_2_ values, even for high acceleration (×5). The T_2_ values for the last tube (T_2_ = 250ms) were out of range (> 300 ms) for the 2D T_2_p‐SSFP sequence and therefore are not shown

The accuracy of the T_2_ values obtained with the proposed 3D MUST‐T_2_ approach in phantom is shown in Figure 3 for different acceleration factors. Excellent linear correlation was found between 3D MUST‐T_2_ and the ground‐truth T_2_ values obtained with SE, with high coefficients of determination (R^2^ > 0.99) for all acceleration factors (×1 to ×5), suggesting high accuracy of the proposed 3D sequence even for high acceleration factors. Lower linear correlation (R^2^ = 0.95) was observed for conventional 2D T_2_p‐SSFP in comparison to reference SE measurements. Bias between SE and 2D T_2_p‐SSFP with linear order acquisitions has been reported previously.^3^

Bland‐Altman analysis demonstrated good T_2_ agreement for all 31 slices of 3D MUST‐T_2_ (Supporting Information Figure S5). The mean differences with 95% confidence interval (CI) between 3D MUST‐T_2_ and SE were 0.6 ms (95% CI, −2.1 ms to 0.89 ms) for short T_1_ myocardium ([T_2_,T_1_] = [48,803] ms), 0.6 ms (95% CI, −3.7 to 2.5 ms) for medium T_1_ myocardium ([T_2_,T_1_] = [48,1090] ms), and 1.4 ms (95% CI, −4.9 ms to ‐2.0 ms) for long T_1_ myocardium ([T_2_,T_1_] = [50,1333] ms).

### 3.3 In vivo experiments

All healthy subject and patient acquisitions/reconstructions were performed successfully, and analysis results are reported hereafter.

#### 3.3.1 Healthy subjects

**Figure 4:**
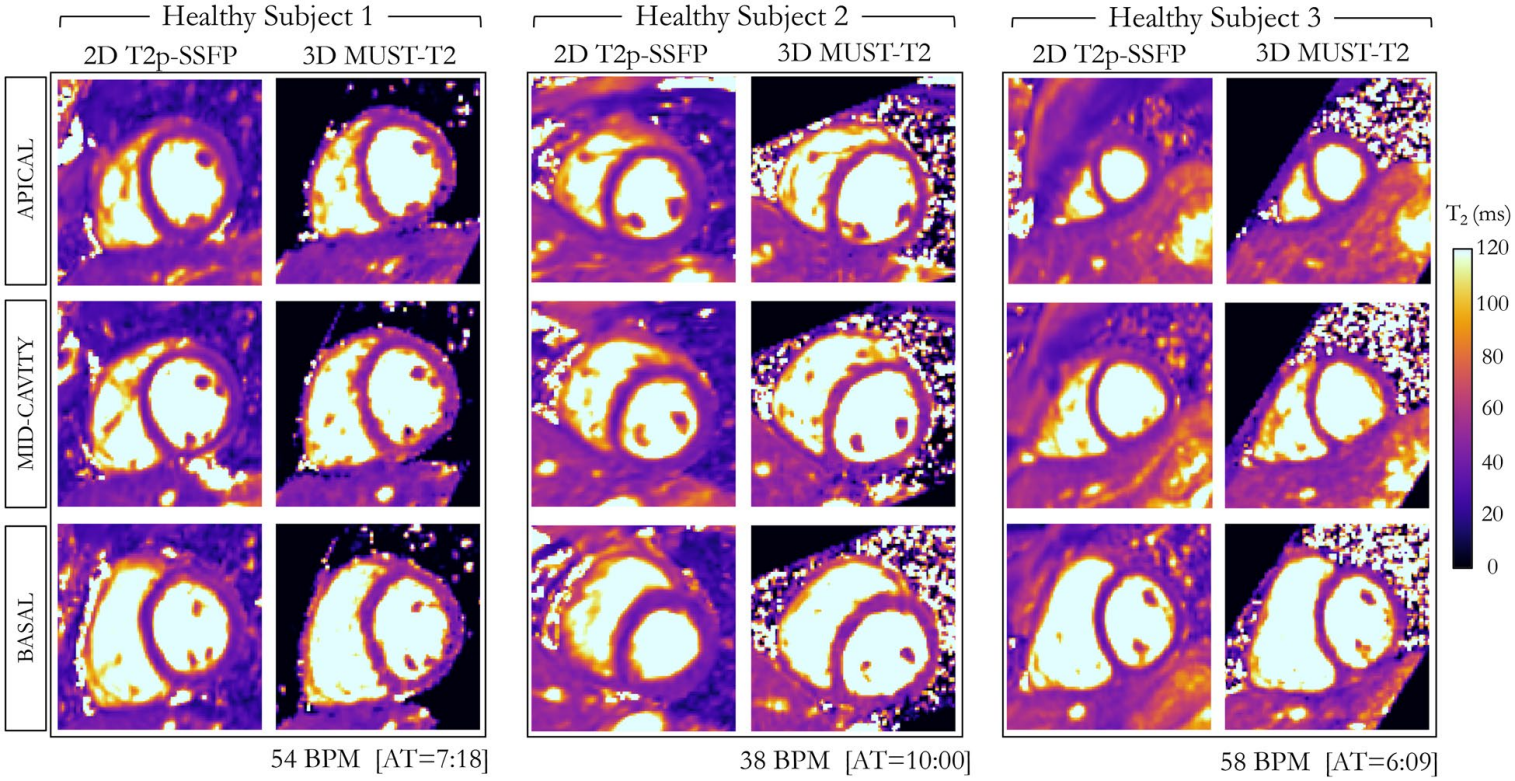
The T_2_ maps obtained using the proposed free‐breathing 3D MUST‐T_2_ sequence and the conventional breath‐held 2D T_2_p‐SSFP sequence are shown for 3 healthy subjects. The 3D MUST‐T_2_ slices were reformatted to short axis to match the 2D T_2_ map acquisitions. Good visualization of the myocardium and surrounding structures can be observed on the 3D MUST‐T_2_ maps. Acquisition times are expressed as minutes:seconds. Abbreviations: AT, acquisition time

The average acquisition time of the proposed free‐breathing 3D MUST‐T_2_ sequence in healthy subjects was 6 minutes 43seconds ± 1 minute 37 seconds (range 4‐10 minutes, heart rate range: 38‐85 bpm) with 100% scan efficiency. Translational motion estimation and correction was performed in about 20 seconds, whereas the average reconstruction time (including HD‐PROST reconstruction and dictionary matching) was about 3 minutes. Representative T_2_ maps from 3 healthy subjects acquired with the proposed 3D MUST‐T_2_ sequence and the conventional 2D T_2_p‐SSFP sequence are shown in Figure 4. Both free‐breathing 3D MUST‐T_2_ and breath‐held 2D T_2_p‐SSFP show comparable visualization of the left ventricle and surrounding structures (e.g., papillary muscles). Reconstructed T_2_ maps from additional healthy subjects are shown in Supporting Information Figure S6 and Supporting Information Figure S7.

**Figure 5:**
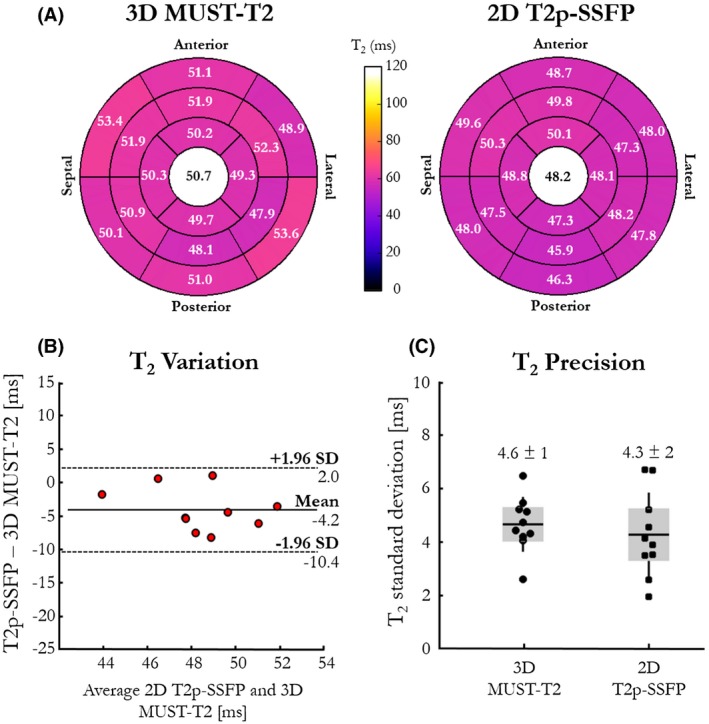
Accuracy and precision of the proposed 3D MUST‐T_2_ mapping sequence. A, The T_2_ accuracy of the proposed 3D MUST‐T_2_ sequence versus conventional 2D T_2_p‐SSFP, as measured by the mean T_2_ value, are shown in the left ventricular segmentation. The T_2_ values are in good agreement with the literature (T_2_ = 50 ± 4 ms^33^). The averaged T_2_ relaxation times over the whole myocardium are shown in the bull's eye plots' center. Accuracy (B) and precision (C) of T_2_ relaxation times (ms) obtained in the myocardial septum with the proposed 3D MUST‐T_2_ and the conventional 2D T_2_p‐SSFP are shown for the 10 healthy subjects

Bull's eye plots based on the American Heart Association's 16‐segment model of the left ventricle obtained with 3D MUST‐T_2_ and 2D T_2_p‐SSFP mappings are depicted in Figure 5A. A small, although significant, overestimation of regional T_2_ values over the whole myocardium was observed with 3D MUST‐T_2_ in comparison to 2D T_2_p‐SSFP mapping (50.7 ± 1.7 ms for 3D MUST‐T_2_ versus 48.2 ± 1.3 ms for 2D T_2_p‐SSFP, *P* < .05). We found no statistical differences in regional T_2_ values between segments with 3D MUST‐T_2_ for all subjects (*P* = .6). A small overestimation of septal T_2_ values was observed with 3D MUST‐T_2_ in comparison to 2D T_2_p‐SSFP mapping with linear k‐space reordering (mean difference: −4.2 ms, ±95% confidence interval: −10.4/2.0 ms, Figure 5B). Precision of T_2_ values was similar for both techniques (5 ± 1 ms for 3D MUST‐T_2_ versus 4 ± 2 ms for 2D T_2_p‐SSFP, *P* = .520, Figure 5C). The corresponding average coefficient of variation for the proposed 3D MUST‐T_2_ approach was similar to that of the 2D conventional sequence (9.13% versus 9.09%, respectively, *P* = .097). There were no statistical differences in mean T_2_ values with 3D MUST‐T_2_ among basal, midventricular, and apical slices for all subjects (*P* = .655).

The effect of heart‐rate differences across healthy subjects on T_2_ values is shown in Figure 2D. The T_2_ measurements obtained from 3D MUST‐T_2_ in vivo showed no significant correlation with heart rate (T_2_ = 0.026 × *heart rate* + 49 ms, *P* = .919).

**Figure 6:**
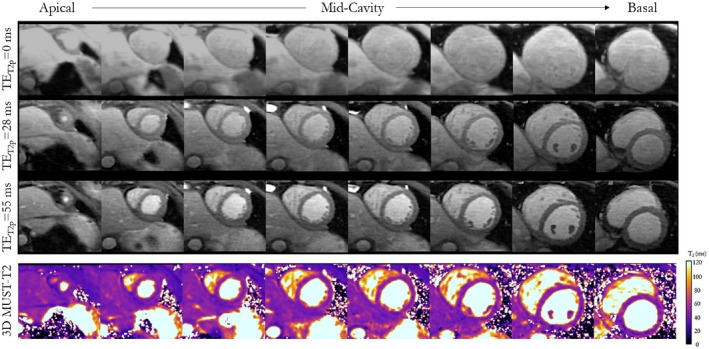
Three‐dimensional visualization of the acquired T_2_‐weighted images and the corresponding T_2_ volume. Representative T_2_‐weighted images for subject 2 (acquisition time: 10 minutes, heart rate = 38 bpm), and the corresponding T_2_ maps obtained by the proposed 3D MUST‐ T_2_. Eight reformatted short‐axis slices that cover the heart from apex to base are shown. Uniform distribution of T_2_ values through the slices over the whole left ventricle can be observed. The color scale indicates T_2_ values between 0 ms and 120 ms

Eight short‐axis slices of the left ventricular myocardium for 1 representative healthy subject obtained with the proposed 3D MUST‐T_2_ approach are shown in Figure 6. The reconstructed T_2_‐weighted images and the corresponding T_2_ maps are included. No noticeable residual undersampling artifacts were observed on the native T_2_‐weighted images the and corresponding T_2_ maps for the entire left ventricle.

**Figure 7:**
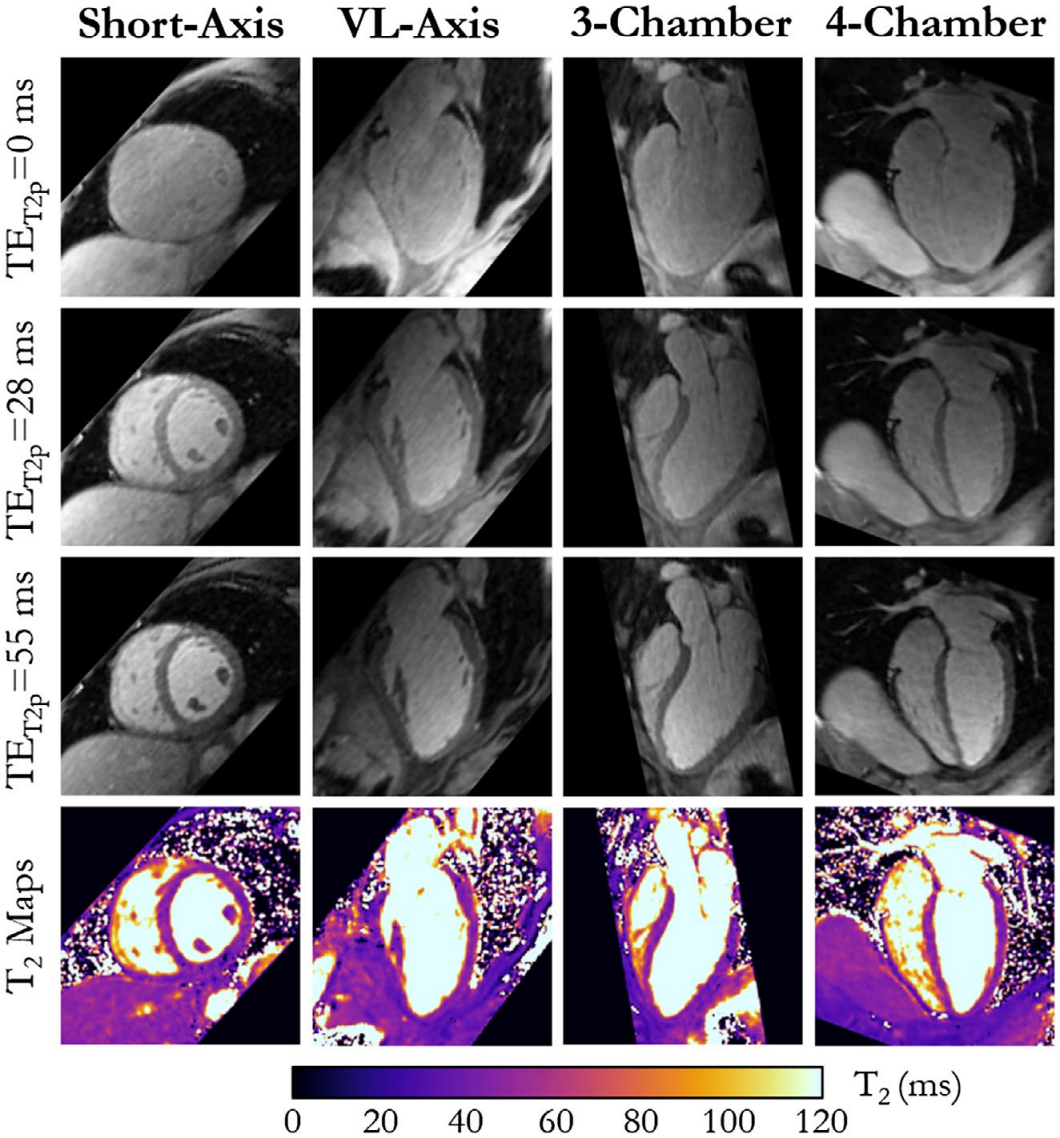
Representative T_2_‐prepared images for subject 2 and the corresponding T_2_ maps obtained with the proposed 3D MUST‐T_2_ sequence. Reformats in short‐axis, vertical long‐axis (VL), 3‐chamber, and 4‐chamber views are shown

Because MUST‐T_2_ acquisitions were performed with isotropic spatial resolution and whole‐heart coverage, the T_2_ maps can be reformatted in any arbitrary plane. This advantage is best appreciated in Figure 7, where reformats in multiple standard orientations (short‐axis, vertical long‐axis, 3‐chamber, and 4‐chamber) are shown for a healthy subject.

The need for using 2D iNAVs to correct for respiratory motion is shown in Supporting Information Figure S8, where the T_2_ maps of 2 healthy subjects are shown with and without 2D translational motion correction. Motion‐corrected T_2_ maps result in better visualization of the myocardium as compared with the non‐motion‐corrected T_2_ maps, which show some spatial blurring.

#### 3.3.2 Patients

**Figure 8:**
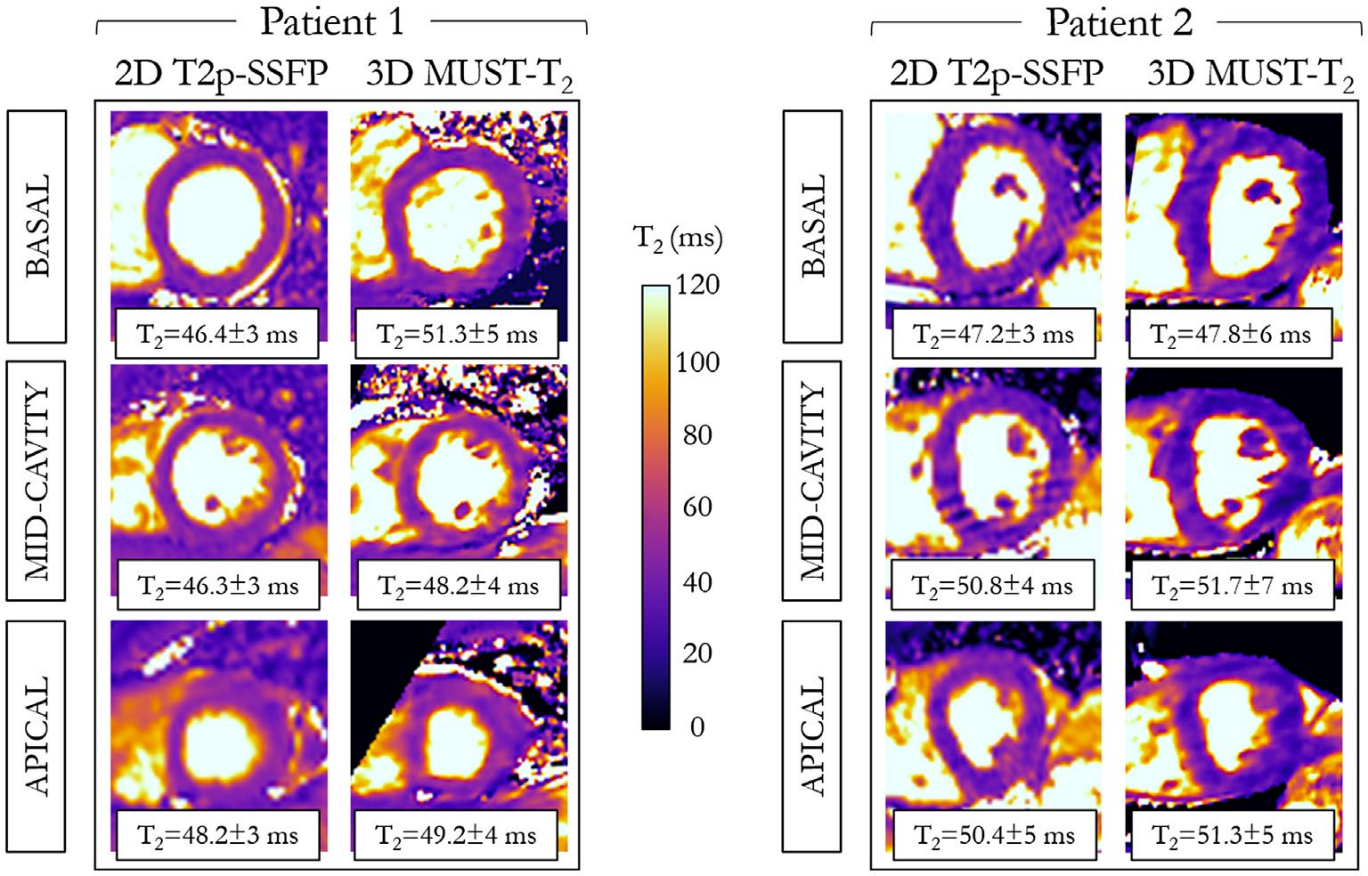
Short‐axis T_2_ maps at apical, midventricular, and basal level for 2 patients acquired with the proposed free‐breathing 3D MUST‐T_2_ framework and the conventional breath‐hold 2D T_2_p‐SSFP sequence. The septal T_2_ relaxation times for each slice are reported as mean ± SD

No cardiac findings were observed in the patient study. The acquisition times of the proposed 3D MUST‐T_2_ sequence were 7 minutes 23 seconds (patient 1, mean heart rate: 75 bpm) and 8 minutes 43 seconds (patient 2, mean heart rate: 65 bpm). Figure 8 depicts the reconstructed apical, midcavity, and basal T_2_ map slices for the 2 patients, using the proposed 3D MUST‐T_2_ framework compared with the current clinical reference maps (breath‐held 2D T_2_p‐SSFP). The proposed 3D MUST‐T_2_ produces T_2_ maps with visual appearance comparable to the conventional 2D T_2_p‐SSFP technique. The mean septal T_2_ relaxation times were similar between the 2 techniques (midventricular slice: patient 1: 48.2 ± 4 ms 3D MUST‐T_2_ versus 46.3 ± 3 ms 2D T_2_p‐SSFP, and patient 2: 51.7 ± 7 ms 3D MUST‐T_2_ versus 50.8 ± 4 ms 2D T_2_p‐SSFP).

## 4 DISCUSSION

In this study, we proposed a framework for highly accelerated free‐breathing whole‐heart T_2_ mapping that combines 2D translational respiratory motion correction with a golden‐angle variable‐density spiral‐like Cartesian trajectory and a recently introduced HD‐PROST reconstruction. The proposed 3D MUST‐T_2_ approach enables accurate and precise high‐resolution isotropic (1.5 mm^3^) 3D whole‐heart T_2_ mapping acquisitions in a fast and predictable scan time.

The performance of the proposed 3D T_2_ mapping framework was assessed in simulations, phantom experiments, and 10 healthy subjects, while initial clinical feasibility was demonstrated in 2 patients with suspected cardiovascular disease.

Three‐dimensional MUST‐T_2_ uses a saturation pulse to reset the magnetization immediately after the R‐wave, as proposed by Ding et al,^17^ and therefore is mostly independent of heart‐rate variations. Another advantage of using saturation pulses compared with other T_2_ mapping techniques^3^, ^5^, ^16^, ^31^ is that recovery periods between subsequent heartbeats are not needed, thus reducing the total acquisition time.

Further acceleration through undersampling is crucial to achieve high isotropic resolution in a clinically feasible scan time. In phantom experiments, we observed that acceleration factors of up to 5‐fold led to accurate T_2_ values (R^2^ > 0.99) with respect to reference SE measurements. Besides the use of an efficient undersampling trajectory, the acquisition of low‐resolution 2D iNAVs at every heartbeat also permits a substantial reduction in scan time by allowing 100% respiratory scan efficiency (no data rejection) and predictable scan time,^24^ as opposed to other respiratory‐gated T_2_ mapping techniques based on diaphragmatic navigator‐gated acquisition.^17^ Other free‐breathing T_2_ mapping techniques have been proposed to allow for 100% scan efficiency and predictable scan time. Van Heeswijk et al^5^ proposed a respiratory self‐navigated 3D sequence to acquire isotropic 3D T_2_ maps with resolution of 1.7 mm^3^ in a predictable scan time of about 18 minutes at 3 T, while Basha et al^32^ proposed a 2D multislice sequence with slice tracking. The significant scan‐time reduction achieved with 3D MUST‐T_2_ was exploited to acquire in vivo isotropic whole‐heart Cartesian T_2_ maps in clinically feasible scan times (about 8 minutes), whereas a fully sampled acquisition would have required more than 35 minutes. The total imaging time for 3D MUST‐T_2_ was affected primarily by the subject's heart rate and the number of slices needed to cover the whole heart in the anterior–posterior direction. For comparison, the conventional breath‐hold 2D T_2_p‐SSFP sequence would have required between 5 and 7 minutes to cover the entire heart (assuming 7‐second breathing commands, 10‐second resting time between acquisitions, 10‐second breath‐hold, and assuming that 11 to 16 slices are needed to cover the heart). The proposed 3D T_2_ mapping sequence can also be performed with lower anisotropic resolution (e.g., 2.0 × 2.0 × 6.0 mm^3^ as achieved in Yang^16^), which will result in shorter scan times and inherently co‐registered slices due to the 3D nature of the acquisition.

The acquisition of undersampled data comes, however, at the cost of significant loss of information that needs to be recovered. Multicontrast HD‐PROST reconstruction takes advantage of the local, nonlocal, and contrast redundancies found in the T_2_‐weighted images, to recover whole‐heart T_2_ maps with negligible remaining aliasing artifacts. The measurements of myocardial T_2_ values by 3D MUST‐T_2_ in healthy subjects (average septal T_2_ = 50.8 ± 3 ms) were in good agreement with in vivo values published in a previous study at 1.5 T with centric ordering (T_2_ = 50 ± 4 ms^33^). The T_2_ overestimation with respect to the conventional breath‐hold 2D T_2_p‐SSFP sequence was observed, with a bias of 4.2 ± 6.2 ms. This overestimation was likely due to the choice of centric (3D MUST‐T_2_) versus linear (2D T_2_p‐SSFP) k‐space ordering, as observed by Giri et al.^3^ Other confounding factors such as heart‐rate variations^34^ not present in the phantom study, or the use of fat saturation and adiabatic T_2_ preparation pulses, could also explain this overestimation in vivo and will be explored further.

Although high acceleration factors were reached with the proposed 3D sequence, precision of T_2_ values in vivo did not show significant deviations from the conventional 2D T_2_p‐SSFP acquisitions (4.6 ± 1 ms versus 4.3 ± 2 ms, *P* = .520) and were similar to previously reported values at 3 T.^5^, ^17^ This can be attributed to the good performance of HD‐PROST reconstruction in concert with the variable‐density trajectory with spiral profile order trajectory, which limits the propagation of noise in the T_2_‐weighted images, noise being a severe side effect of saturation‐based MR mapping techniques.^16^, ^35^, ^36^

Dictionary‐based matching was used in this study to accurately estimate myocardial T_2_ values by pregenerating a dictionary representative of the proposed T_2_ mapping sequence, and whose elements are composed of simulated signal evolution curves.^28^ Our findings in phantom experiments showed that dictionary‐based matching has the potential to accurately estimate myocardial T_2_ values acquired with both 3D MUST‐T_2_ and conventional T_2_p‐balanced SSFP, whereas a T_2_ bias was observed with conventional mono‐exponential fitting for both methods (Supporting Information Figure S4). This can be partly explained by the fact that mono‐exponential fitting does not accurately describe the acquisition parameters and the signal evolution during acquisition, such as balanced SSFP T_1_/T_2_ ratio of tissue, subject‐specific heart rate, number of segments per heartbeat, and number of linear ramp‐up pulses. Therefore, the use of dictionary‐based matching could also improve conventional 2D T_2_p‐SSFP and potentially reduce the bias observed with linear phase encoding.

A limitation of the current framework is that only 2D translation respiratory motion correction was performed to ensure fast reconstruction times. It has been shown in previous reports that nonrigid motion registration can improve reproducibility and spatial variability of 2D T_2_ mapping techniques.^8^, ^11^ Although this issue was not shown to be significant in the present experiments, it can be overcome by incorporating 3D nonrigid respiratory motion correction directly in the image reconstruction.^37^, ^38^, ^39^ Another alternative to deal with large motion between the T_2_‐weighted images is to extend the proposed HD‐PROST reconstruction to enable motion‐resolved reconstruction by extending the searching neighborhood for patch selection to the spatial‐temporal dimension.^40^

In this study, middiastolic imaging was used to minimize cardiac motion and guarantee long saturation times. However, in subjects with highly variable heart rates or high heart rates, end‐systolic imaging may be preferred. Future studies will investigate the effect of shorter saturation times on the accuracy and precision of saturation‐based T_2_ mapping; however, our phantom experiments demonstrate that accuracy is not affected for different heart rates between 50 and 100 bpm.

Moreover, incomplete saturation of fat signal can occur in some regions of the T_2_‐weighted images, especially around the epicardial fat area. This effect can lead to fat‐myocardium partial volume effect, apparent distortion, and loss of sharpness on the T_2_ map and may alter the T_2_ measurements. Further optimized fat suppression for each T_2_‐weighted image will be investigated in the future to alleviate this difficulty.

Translational motion estimation and correction was performed inline in the scanner software, while HD‐PROST reconstruction and dictionary matching were performed offline. The efficient multithreaded implementation of HD‐PROST allowed for fast T_2_ map reconstruction (in the order of 3 minutes). Further speedup to reach subminute runtime could be achieved by implementing the reconstruction on multiple GPUs and using coil compression strategies.^41^

It should be noted that 3D MUST‐T_2_ is based on a T_2_‐prepared sequence that is used commonly in coronary MRA.^42^ The acceptable contrast provided by the last T_2_‐weighted volume (TE_T2prep_ = 55 ms, Figures 6 and 7) could also be exploited to visualize whole‐heart cardiac and proximal coronary artery anatomy. Thus, 3D MUST‐T_2_ may be used for the simultaneous assessment of cardiac and coronary anatomy and myocardial T_2_ relaxation times in a highly simplified and efficient single free‐breathing acquisition. This additional gain could potentially benefit patients with acute non‐ST‐segment elevation myocardial infarction, where obstructive coronary artery disease and myocardial edema are often characteristic.^13^

The proposed free‐breathing T_2_ mapping framework could benefit many cardiovascular patients with severe shortness of breath and where imaging under breath‐holding is often challenging. Preliminary insights into the potential of the proposed 3D MUST‐T_2_ mapping technique in a clinical setting was provided for 2 patients with suspected cardiovascular disease. Although no pathologies were observed in this proof‐of‐concept study, scanning patients often leads to new challenges (e.g., variable heart rate, irregular breathing pattern, poor compliance), resulting in degraded image quality. Higher amplitude in respiratory motion of the heart, as provided by the 2D iNAVs, was observed in patients compared with healthy controls (Supporting Information Figure S9). The mean septal T_2_ values measured on the 2 patients were comparable to that of the healthy subjects. Besides T_2_ relaxation times conforming to the literature (Figure 8), the 3D whole‐heart isotropic coverage of the proposed technique offers the opportunity to reformat the T_2_ maps in any desired orientation, which can be particularly useful for the comprehensive assessment of pathological tissues with complex geometry.^6^

The proposed framework also shares similarities with the recent qBOOST‐T_2_ approach^43^ that enables co‐registered bright‐blood and black‐blood whole‐heart imaging, together with T_2_ quantification and coronary lumen visualization, through the use of an inversion pulse every 3 heartbeats. Although the latest technique may be preferred whenever coronary assessment or precontrast black‐blood images are clinically required, a thorough comparison of the 2 approaches in large patient cohorts will need to be studied.

Finally, the proposed fast and efficient framework holds promise for wider cardiac applications, such as high‐resolution motion‐compensated 3D T_1_ mapping and 3D joint T_1_‐T_2_ mapping,^44^, ^45^, ^46^, ^47^, ^48^, ^49^ both of which will be investigated in future work.

## 5 CONCLUSIONS

A novel approach was developed to enable free‐breathing whole‐heart 3D T_2_ mapping with high isotropic spatial resolution (1.5 mm^3^) in a clinically feasible scan time (< 8 minutes with 100% scan efficiency and predictable scan time). The proposed 3D MUST‐T_2_ framework achieved accurate T_2_ quantification in phantom and in vivo with fast acquisition. Three‐dimensional MUST T_2_ mapping may have the potential to aid the management of many cardiomyopathies in which fast and efficient free‐breathing acquisitions are key to patient comfort, whereas high isotropic resolution is crucial to accurately map tissue heterogeneity. Further studies to assess the clinical utility of 3D MUST‐T_2_ mapping in patients with myocardial inflammation are now warranted.

## CONFLICT OF INTEREST

Dr. Radhouene Neji is employed by Siemens Healthcare, Frimley, United Kingdom.

## Supporting information


**FIGURE S1** A 3D Cartesian variable‐density trajectory was used to allow for fast acquisition of multiple T_2_‐weighted images. The Cartesian trajectory with spiral order samples the k_y_‐k_z_ phase‐encoding plane following approximate spiral interleaves on the Cartesian grid with variable density along each spiral arm. In this sketch, the 2 first acquired spirals are shown for each contrast (each spiral containing 20 segments). A golden angle rotation between successive spirals and successive contrasts is applied to introduce incoherently distributed aliasing artifacts along the contrast dimension, and noise‐like artifacts in the spatial dimension
**FIGURE S2** Flowchart of the optimization 2 of the proposed HD‐PROST. Denoising of multiple T_2_‐weighted images is performed using a 3D block matching, which groups and unfolds similar 3D patches in the noisy multicontrast images to form a low‐rank 2D matrix. A third‐order tensor is formed by stacking the T_2_ contrast dimension on the third dimension. The high‐order tensor of size N (number of pixels in each patch) × K (number of similar patches within a neighborhood) × L (number of T_2_ contrasts) admits a low multilinear rank approximation and can be compressed through high‐order tensor decomposition by truncating the multilinear singular vectors that correspond to small multilinear singular values. The outputs of this step are the denoised multicontrast images that are then used in the joint regularized reconstruction step (optimization 1) as prior knowledge. Reconstruction parameters used in this study are shown (bottom row). We refer the reader to Bustin et al^18^ for more information on these parameters. Reconstruction parameter details: L, number of contrasts; K, number of similar patches; N, patch size (in pixels); *λ* *_p_*, threshold value
**FIGURE S3** Simulations of the proposed T_2_ mapping sequence were performed using the EPG formalism to assess the effect of T_1_ in the EPG‐based dictionary on the matched T_2_ value. Signal evolutions for different T_1_ (from 850 to 1200 with a step size of 50 ms) and T_2_ (from 42 to 82 with a step size of 8 ms) were generated and matched to a dictionary simulated with fixed T_1_ (1100 ms) and varying T_2_s (similar to the one used in the phantom experiment). A, Matched T_2_ is plotted as a function of T_1_. B, The signal evolutions corresponding to short (T_1_/T_2_ = 800/52 ms), medium (T_1_/T_2_ = 1100/52 ms), and long (T_1_/T_2_ = 1300/52 ms) T_1_ myocardium were generated for the proposed sequence through EPG simulation. The obtained signal evolutions did not appear to differ, suggesting that the proposed 3D MUST‐T_2_ map sequence with dictionary‐based matching is independent of the T_1_ used in the EPG‐based dictionary (matched T_2_s were the same for the 3 generated signals and equal to 52 ms). Therefore, for the phantom and in vivo experiments, we kept the T_1_ constant at 1100 ms
**FIGURE S4** Conventional mono‐exponential fitting and dictionary‐based matching for T_2_ T_2_p‐SSFP T_2_ mapping in comparison to the proposed 3D MUST‐T_2_ sequence for the phantom study. The proposed 3D acquisition with mono‐exponential fitting is also included for comparison purposes. Accurate phantom T_2_ values, in agreement with reference spin echo, were obtained with the proposed 3D MUST‐T_2_ sequence with dictionary‐based matching; however, bias is observed with the proposed acquisition when mono‐exponential fitting is used. Bias is also observed with the conventional (linear phase encoding) 2D T_2_p‐SSFP mapping with mono‐exponential fitting; however, this bias is significantly reduced when dictionary‐based matching is used
**FIGURE S5** Spatial T_2_ uniformity over the slice direction is assessed for the phantom study for 3 vials (corresponding to short, medium, and long T_1_ myocardium). The solid line is the average difference between gold‐standard spin echo and the proposed 3D MUST‐T_2_ mapping sequence, and the dashed lines represent the mean ± 2 SDs between the 2 techniques. Good T_2_ uniformity can be observed with the proposed technique. The mean difference in T_2_ for the vial corresponding to short T_1_ myocardium was −0.6 ms [±95% confidence interval [CI] = −2.1/0.89 ms), −0.6 ms [±95% CI = −3.7/2.5 ms) for the medium T_1_ myocardium, and −1.4 ms [±95% CI = −4.9/2.0 ms) for the long T_1_ myocardium
**FIGURE S6** The T_2_ maps obtained using the proposed free‐breathing 3D MUST‐T_2_ mapping sequence and the conventional breath‐held 2D T_2_p‐SSFP sequence are shown for 3 additional healthy subjects. The 3D MUST‐T_2_ slices were reformatted to short axis to match the 2D T_2_ map acquisitions. Representative 16 American Heart Association segments are shown to illustrate how much spatial information was considered for T_2_ calculation. Acquisition times are expressed as minutes:seconds
**FIGURE S7** Axial view of T_2_ maps acquired using the proposed 3D T_2_ mapping sequence on 3 healthy subjects. Number of slices was adjusted per subject to cover the left ventricle in the anterior–posterior direction
**FIGURE S8** Impact of iNAV‐based beat‐to‐beat translation motion correction on 3D MUST‐T_2_ map is shown for 2 healthy subjects. A, Reconstructed T_2_‐weighted images (TE_T2pr_ _ep_ = 55 ms) are shown before and after motion correction with the corresponding T_2_ maps. Better visualization of the myocardium can be observed after motion correction with clear delineation of cardiac structures and myocardial walls. Note the blurring observed on the non‐motion‐corrected T_2_ maps. B, Plots showing the intensity profiles taken on the T_2_‐weighted images through the heart–liver interface
**FIGURE S9** A, Foot–head respiratory displacements of the heart obtained from the 2D image navigators at each heartbeat are shown for 2 representative healthy subjects (left) and 2 patients (right). The end‐expiration position is used as reference for translational motion estimation. Although regular breathing patterns can be observed on the healthy subjects, more irregular breathing patterns with strong motion amplitudes are observed on patients 1 and 2. B, Average R‐R intervals are shown for each healthy subject and patient. Patient 1 presented with irregular cardiac rhythm (R‐R = 838 ± 211 ms)

## References

[1] Guo H , Au W‐Y , Cheung JS , et al. Myocardial T2 quantitation in patients with iron overload at 3 Tesla. J Magn Reson Imaging. 2009;30:394–400. 19629983 10.1002/jmri.21851 PMC2946793

[2] He T , Gatehouse PD , Anderson LJ , et al. Development of a novel optimized breathhold technique for myocardial T2 measurement in thalassemia. J Magn Reson Imaging. 2006;24:580–585. 16892203 10.1002/jmri.20681

[3] Giri S , Chung Y‐C , Merchant A , et al. T2 quantification for improved detection of myocardial edema. J Cardiovasc Magn Reson. 2009;11:1–13. 20042111 10.1186/1532-429X-11-56 PMC2809052

[4] Haberkorn SM , Spieker M , Jacoby C , Flögel U , Kelm M , Bönner F . State of the art in cardiovascular T2 mapping: on the way to a cardiac biomarker? Curr Cardiovasc Imaging Rep. 2018;11.

[5] Van Heeswijk RB , Piccini D , Feliciano H , Hullin R , Schwitter J , Stuber M . Self‐navigated isotropic three‐dimensional cardiac T2 mapping. Magn Reson Med. 2015;73:1549–1554. 24809849 10.1002/mrm.25258

[6] Ding H , Schar M , Zviman MM , Halperin HR , Beinart R , Herzka DA . High‐resolution quantitative 3D T2 mapping allows quantification of changes in edema after myocardial infarction. J Cardiovasc Magn Reson. 2013;15:345–347.

[7] Van Heeswijk RB , Piccini D , Tozzi P , et al. Three‐dimensional self‐navigated T2 mapping for the detection of acute cellular rejection after orthotopic heart transplantation. Transplant Direct. 2017;3. 10.1097/TXD.0000000000000635 PMC5381742 28405605

[8] Roujol S , Basha TA , Weingartner S , et al. Motion correction for free breathing quantitative myocardial T2 mapping: impact on reproducibility and spatial variability. J Cardiovasc Magn Reson. 2015;17:W5. 10.1186/s12968-015-0141-1 PMC4465156 26067275

[9] Jin N , Jolly M‐P , Raman SV , Simonetti OP . Free‐breathing myocardial T2 mapping using GRE‐EPI and automatic non‐rigid motion correction. J Cardiovasc Magn Reson. 2015;17. 10.1186/s12968-015-0216-z PMC4690363 26699850

[10] Odille F , Escanyé JM , Atkinson D , Bonnemains L , Felblinger J . Nonrigid registration improves MRI T2 quantification in heart transplant patient follow‐up. J Magn Reson Imaging. 2015;42:168–174. 25180788 10.1002/jmri.24741

[11] Giri S , Shah S , Xue H , et al. Myocardial T2 mapping with respiratory navigator and automatic nonrigid motion correction. Magn Reson Med. 2012;68:1570–1578. 22851292 10.1002/mrm.24139 PMC4512252

[12] Rauhalammi S , Layland J , Carrick D , et al. T1 and T2 mapping have higher diagnostic accuracy for the ischaemic area‐at‐risk in NSTEMI patients compared with dark blood imaging. Heart. 2014;100.

[13] Tessa C , Del Meglio J , Lilli A , et al. T1 and T2 mapping in the identification of acute myocardial injury in patients with NSTEMI. Radiol Med. 2018;123:926–934. 30132183 10.1007/s11547-018-0931-2

[14] Puntmann VO , Isted A , Hinojar R , Foote L , Carr‐White G , Nagel E . T1 and T2 mapping in recognition of early cardiac involvement in systemic sarcoidosis. Radiology. 2017;285:63–72. 28448233 10.1148/radiol.2017162732

[15] Van Heeswijk RB , Feliciano H , Bongard C , et al. Free‐breathing 3T magnetic resonance T2‐mapping of the heart. JACC Cardiovasc Imaging. 2012;5:1231–1239. 23236973 10.1016/j.jcmg.2012.06.010

[16] Yang HJ , Sharif B , Pang J , et al. Free‐breathing, motion‐corrected, highly efficient whole heart T2 mapping at 3T with hybrid radial‐cartesian trajectory. Magn Reson Med. 2016;75:126–136. 25753385 10.1002/mrm.25576 PMC4561222

[17] Ding H , Fernandez‐De‐Manuel L , Schär M , et al. Three‐dimensional whole‐heart T2 mapping at 3T. Magn Reson Med. 2015;74:803–816. 25242141 10.1002/mrm.25458

[18] Bustin A , Cruz G , Jaubert O , Lopez K , Botnar RM , Prieto C . High‐dimensionality undersampled patch‐based reconstruction (HD‐PROST) for accelerated multi‐contrast magnetic resonance imaging. Magn Reson Med. 2019;81:3705–3719. 30834594 10.1002/mrm.27694 PMC6646908

[19] Weigel M . Extended phase graphs: dephasing, RF pulses, and echoes—pure and simple. J Magn Reson Imaging. 2015;41:266–295. 24737382 10.1002/jmri.24619

[20] Nezafat R , Stuber M , Ouwerkerk R , Gharib AM , Desai MY , Pettigrew RI . B1‐insensitive T2 preparation for improved coronary magnetic resonance angiography at 3T. Magn Reson Med. 2006;55:858–864. 16538606 10.1002/mrm.20835

[21] Prieto C , Doneva M , Usman M , et al. Highly efficient respiratory motion compensated free‐breathing coronary MRA using golden‐step Cartesian acquisition. J Magn Reson Imaging. 2015;41:738–746. 24573992 10.1002/jmri.24602

[22] Bustin A , Ginami G , Cruz G , et al. Five‐minute whole‐heart coronary MRA with sub‐millimeter isotropic resolution, 100% respiratory scan efficiency, and 3D‐PROST reconstruction. Magn Reson Med. 2019;81:102–115. 30058252 10.1002/mrm.27354 PMC6617822

[23] Winkelmann S , Schaeffter T , Koehler T , Eggers H , Doessel O . An optimal radial profile order based on the golden ratio for time‐resolved MRI. IEEE Trans Med Imaging. 2007;26:68–76. 17243585 10.1109/TMI.2006.885337

[24] Henningsson M , Koken P , Stehning C , Razavi R , Prieto C , Botnar RM . Whole‐heart coronary MR angiography with 2D self‐navigated image reconstruction. Magn Reson Med. 2012;67:437–445. 21656563 10.1002/mrm.23027

[25] Ginami G , Neji R , Phinikaridou A , Whitaker J , Botnar RM , Prieto C . Simultaneous bright‐ and black‐blood whole‐heart MRI for noncontrast enhanced coronary lumen and thrombus visualization. Magn Reson Med. 2018;79:1460–1472. 28722267 10.1002/mrm.26815 PMC5811778

[26] Bustin A , Voilliot D , Menini A , et al. Isotropic reconstruction of MR images using 3D patch‐based self‐similarity learning. IEEE Trans Med Imaging. 2018;37:1932–1942. 29994581 10.1109/TMI.2018.2807451

[27] Hennig J , Weigel M , Scheffler K . Calculation of flip angles for echo trains with predefined amplitudes with the extended phase graph (EPG)‐algorithm: principles and applications to hyperecho and TRAPS sequences. Magn Reson Med. 2004;51:68–80. 14705047 10.1002/mrm.10658

[28] Roccia E , Vidya Shankar R , Neji R , et al. Accelerated 3D T2 mapping with dictionary‐based matching for prostate imaging. Magn Reson Med. 2019;81:1795–1805. 30368900 10.1002/mrm.27540

[29] Captur G , Gatehouse P , Keenan KE , et al. A medical device‐grade T1 and ECV phantom for global T1 mapping quality assurance—the T1 mapping and ECV standardization in cardiovascular magnetic resonance (T1MES) program. J Cardiovasc Magn Reson. 2016;18:1–20. 27660042 10.1186/s12968-016-0280-z PMC5034411

[30] Cerqueira MD , Weissman NJ , Dilsizian V , et al. Standardized myocardial segmentation and nomenclature for tomographic imaging of the heart: a statement for healthcare professionals from the cardiac imaging. Circulation. 2002;105. 10.1161/hc0402.102975 11815441

[31] Darçot E , Yerly J , Colotti R , et al. Accelerated and high‐resolution cardiac T2 mapping through peripheral k‐space sharing. Magn Reson Med. 2019;81:220–233. 30058085 10.1002/mrm.27374

[32] Basha TA , Bellm S , Roujol S , Kato S , Nezafat R . Free‐breathing slice‐interleaved myocardial T2 mapping with slice‐selective T2 magnetization preparation. Magn Reson Med. 2016;76:555–565. 26479866 10.1002/mrm.25907 PMC4837110

[33] Blume U , Lockie T , Stehning C , et al. Interleaved T1 and T2 relaxation time mapping for cardiac applications. J Magn Reson Imaging. 2009;29:480–487. 19161206 10.1002/jmri.21652

[34] Granitz M , Motloch LJ , Granitz C , et al. Comparison of native myocardial T1 and T2 mapping at 1.5T and 3T in healthy volunteers: reference values and clinical implications. Wien Klin Wochenschr. 2019;131:143–155. 30519737 10.1007/s00508-018-1411-3 PMC6459801

[35] Nordio G , Bustin A , Henningsson M , et al. 3D SASHA myocardial T1 mapping with high accuracy and improved precision. Magn Reson Mater Phys Biol Med. 2019;32:281–289. 10.1007/s10334-018-0703-y PMC6424941 30191345

[36] Bustin A , Ferry P , Codreanu A , et al. Impact of denoising on precision and accuracy of saturation‐recovery‐based myocardial T1 mapping. J Magn Reson Imaging. 2017;46:1377–1388. 28376285 10.1002/jmri.25684

[37] Odille F , Menini A , Escanye J‐M , et al. Joint reconstruction of multiple images and motion in MRI: application to free‐breathing myocardial T2 quantification. IEEE Trans Med Imaging. 2016;35:197–207. 26259015 10.1109/TMI.2015.2463088

[38] Batchelor PG , Atkinson D , Irarrazaval P , Hill D , Hajnal J , Larkman D . Matrix description of general motion correction applied to multishot images. Magn Reson Med. 2005;54:1273–1280. 16155887 10.1002/mrm.20656

[39] Cruz G , Atkinson D , Henningsson M , Botnar RM , Prieto C . Highly efficient nonrigid motion‐corrected 3D whole‐heart coronary vessel wall imaging. Magn Reson Med. 2017;77:1894–1908. 27221073 10.1002/mrm.26274 PMC5412916

[40] Kuestner T , Bustin A , Cruz G , et al. 3D Cartesian free‐running cardiac and respiratory resolved whole‐heart MRI In: Proceedings of the 27th Annual Meeting of ISMRM, Montreal, Canada, 2019 p 2192.

[41] Zhang T , Pauly JM , Vasanawala SS , Lustig M . Coil compression for accelerated imaging with Cartesian sampling. Magn Reson Med. 2013;69:571–582. 22488589 10.1002/mrm.24267 PMC3396763

[42] Botnar RM , Stuber M , Danias PG , Kissinger KV , Manning WJ . Improved coronary artery definition with T2‐weighted, free‐breathing, three‐dimensional coronary MRA. Circulation. 1999;99:3139–3148. 10377077 10.1161/01.cir.99.24.3139

[43] Milotta G , Ginami G , Bustin A , Neji R , Prieto C , Botnar RM . 3D whole‐heart free‐breathing BOOST‐T2 mapping. In: Proceedings of the 27th Annual Meeting of ISMRM, Montreal, Canada, 2019 p 2002.

[44] Akçakaya M , Weingärtner S , Basha TA , Roujol S , Bellm S , Nezafat R . Joint myocardial T1 and T2 mapping using a combination of saturation recovery and T2‐preparation. Magn Reson Med. 2016;76:888–896. 26418119 10.1002/mrm.25975 PMC4811754

[45] Kvernby S , Warntjes M , Haraldsson H , Carlhäll CJ , Engvall J , Ebbers T . Simultaneous three‐dimensional myocardial T1 and T2 mapping in one breath hold with 3D‐QALAS. J Cardiovasc Magn Reson. 2014;16:102. 25526880 10.1186/s12968-014-0102-0 PMC4272556

[46] Xanthis CG , Bidhult S , Greiser A , et al. Simulation‐based quantification of native T1 and T2 of the myocardium using a modified MOLLI scheme and the importance of magnetization transfer. Magn Reson Imaging. 2018;48:96–106. 29288037 10.1016/j.mri.2017.12.020

[47] Guo R , Chen Z , Herzka DA , Luo J , Ding H . A three‐dimensional free‐breathing sequence for simultaneous myocardial T1 and T2 mapping. Magn Reson Med. 2018;81:1031–1043. 30393892 10.1002/mrm.27466

[48] Santini F , Kawel‐Boehm N , Greiser A , Bremerich J , Bieri O . Simultaneous T1 and T2 quantification of the myocardium using cardiac balanced‐SSFP inversion recovery with interleaved sampling acquisition (CABIRIA). Magn Reson Med. 2015;74:365–371. 25113911 10.1002/mrm.25402

[49] Milotta G , Ginami G , Bustin A , Neji R , Prieto C , Botnar RM . 3D whole‐heart high‐resolution motion compensated joint T1/T2 mapping. In: Proceedings of the 27th Annual Meeting of ISMRM, Montreal, Canada, 2019 p 2003.

