# Supplementary material for: Accelerated free‐breathing whole‐heart 3D T2 mapping with high isotropic resolution
Source: Magn Reson Med. 2019 Sep 19;83(3):988–1002. doi: 10.1002/mrm.27989 (PMC6899588; doi:10.1002/mrm.27989)
Supplement: Supplementary file 1 — FIGURE S1 A 3D Cartesian variable‐density trajectory was used to allow for fast acquisition of multiple T2‐weighted images. The Cartesian trajectory with spiral order samples the ky‐kz phase‐encoding plane following approximate spiral interleaves on the Cartesian grid with variable density along each spiral arm. In this sketch, the 2 first acquired spirals are shown for each contrast (each spiral containing 20 segments). A golden angle rotation between successive spirals and successive contrasts is applied to introduce incoherently distributed aliasing artifacts along the contrast dimension, and noise‐like artifacts in the spatial dimension FIGURE S2 Flowchart of the optimization 2 of the proposed HD‐PROST. Denoising of multiple T2‐weighted images is performed using a 3D block matching, which groups and unfolds similar 3D patches in the noisy multicontrast images to form a low‐rank 2D matrix. A third‐order tensor is formed by stacking the T2 contrast dimension on the third dimension. The high‐order tensor of size N (number of pixels in each patch) × K (number of similar patches within a neighborhood) × L (number of T2 contrasts) admits a low multilinear rank approximation and can be compressed through high‐order tensor decomposition by truncating the multilinear singular vectors that correspond to small multilinear singular values. The outputs of this step are the denoised multicontrast images that are then used in the joint regularized reconstruction step (optimization 1) as prior knowledge. Reconstruction parameters used in this study are shown (bottom row). We refer the reader to Bustin et al18 for more information on these parameters. Reconstruction parameter details: L, number of contrasts; K, number of similar patches; N, patch size (in pixels); λ p, threshold value FIGURE S3 Simulations of the proposed T2 mapping sequence were performed using the EPG formalism to assess the effect of T1 in the EPG‐based dictionary on the matched T2 value. Signal evolutions fo [file MRM-83-988-s001.docx]

**
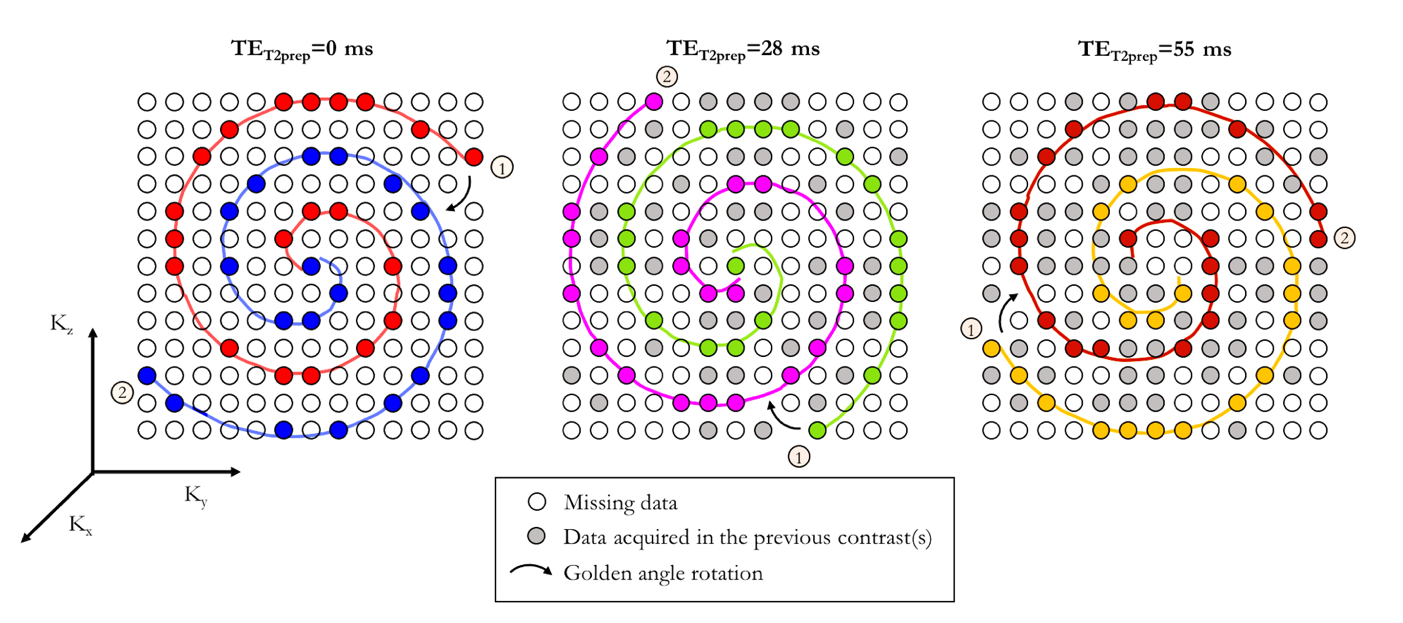
**

**Supporting Information Figure S1** A 3D Cartesian variable-density trajectory was employed to allow for fast acquisition of multiple T2w images. The Cartesian trajectory with spiral order samples the k_y_-k_z_ phase encoding plane following approximate spiral interleaves on the Cartesian grid with variable density along each spiral arm. In this sketch, the 2 first acquired spirals are shown for each contrast, each spiral containing 20 segments. A golden angle rotation between successive spirals and successive contrasts is applied to introduce incoherently distributed aliasing artifacts along the contrast dimension, and noise-like artifacts in the spatial dimension.

**
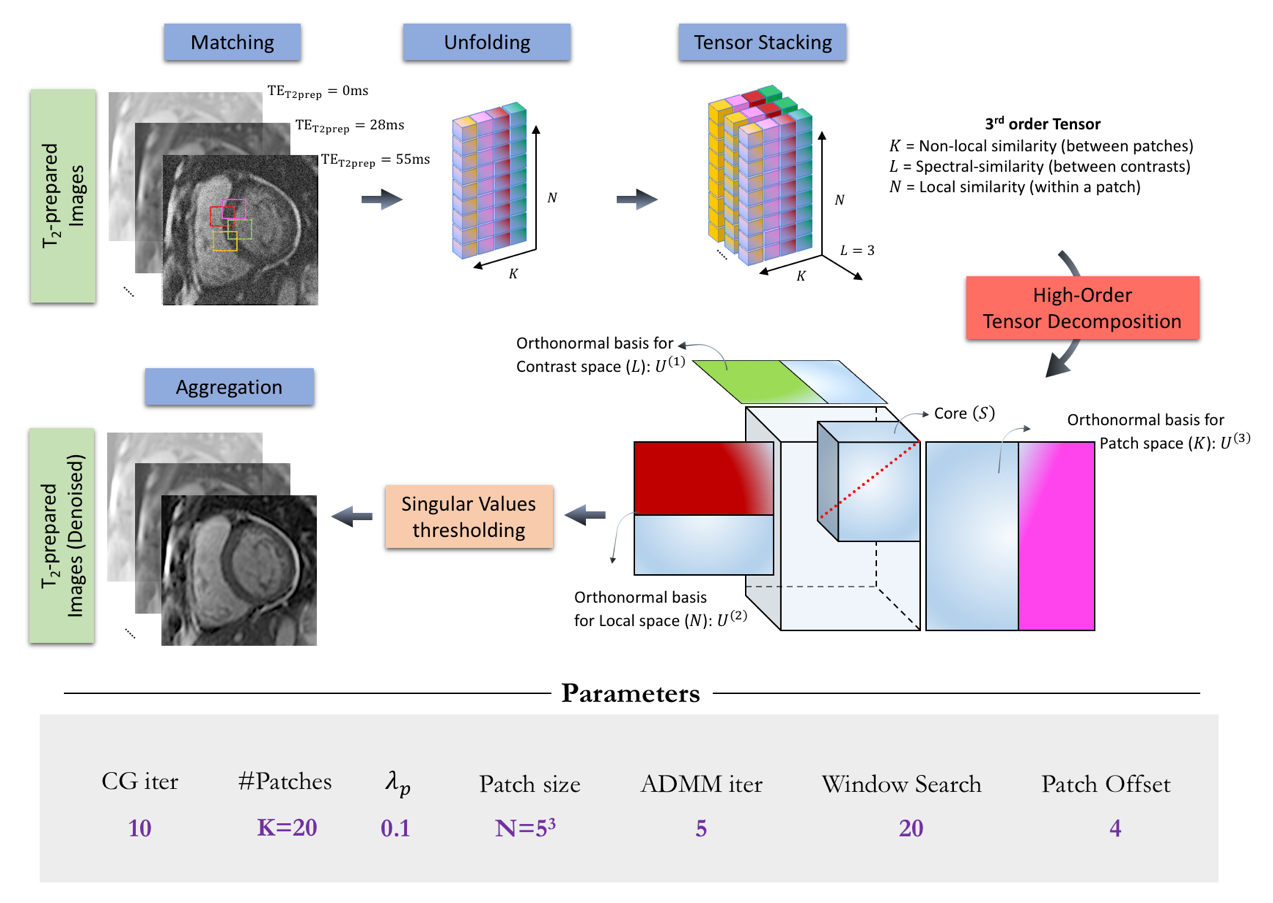
**

**Supporting Information Figure S2** Flowchart of the optimization 2 of the proposed High-Dimensionality Patch-based RecOnSTruction (HD-PROST). Denoising of multiple T2w images is performed using a 3D block matching, which groups and unfolds similar 3D patches in the noisy multi-contrast images to form a low-rank 2D matrix. A third-order tensor is formed by stacking the T2 contrast dimension on the third dimension. The high-order tensor of size N (number of pixels in each patch) x K (number of similar patches within a neighborhood) x L (number of T2 contrasts) admits a low multilinear rank approximation and can be compressed, through high-order tensor decomposition, by truncating the multilinear singular vectors that correspond to small multilinear singular values. The outputs of this step are the denoised multi-contrast images which are then used in the joint regularized reconstruction step (optimization 1) as prior knowledge. Reconstruction parameters used in this study are shown (bottom row). We refer the reader to (18) for more information on these parameters. Reconstruction parameter details: N, patch size (in pixels); L, number of contrasts; K, number of similar patches; $\lambda_{p}$, threshold value.

**
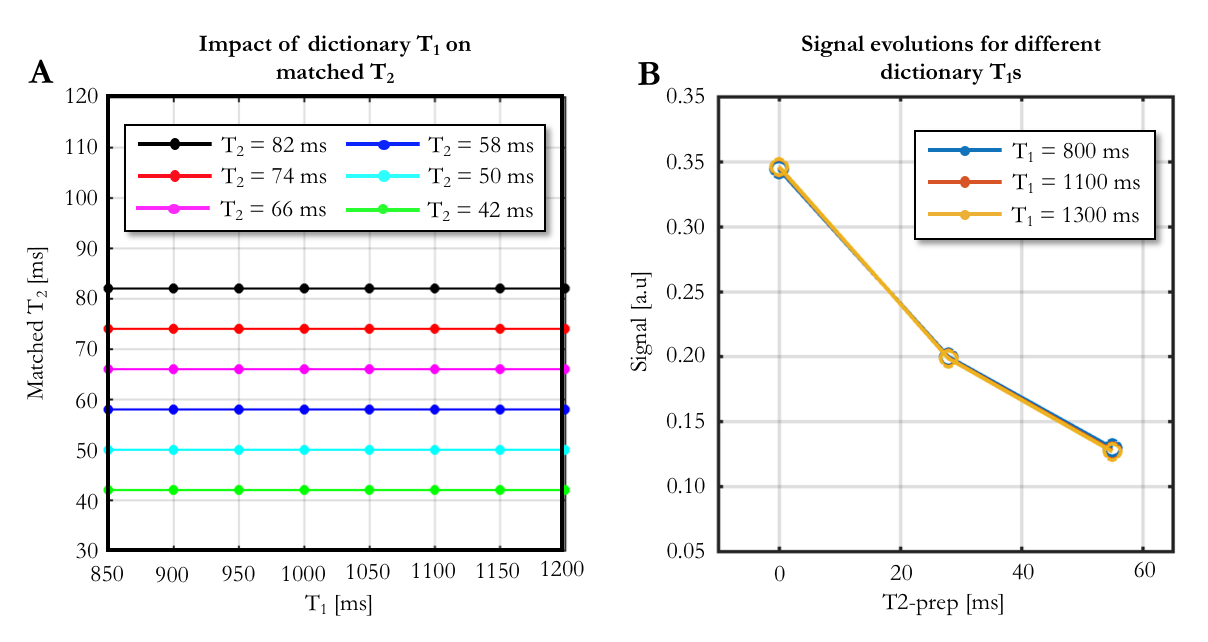
**

**Supporting Information Figure S3** Simulations of the proposed T2 mapping sequence were performed using the EPG formalism to assess the effect of T1 in the EPG-based dictionary on the matched T2 value. Signals evolutions for different T1 (from 850 to 1200 with a step size of 50 ms) and T2 (from 42 to 82 with a step size of 8 ms) were generated and matched to a dictionary simulated with fixed T1 (1100 ms) and varying T2s (similar to the one used in the phantom experiment). (A) Matched T2 is plotted as a function of T1. (B) The signal evolutions corresponding to short (T1/T2 = 800/52 ms), medium (T1/T2 = 1100/52 ms), and long (T1/T2 = 1300/52 ms) T1 myocardium were generated for the proposed sequence through EPG simulation. The obtained signal evolutions did not seem to differ, suggesting that the proposed 3D MUST-T2 map sequence with dictionary-based matching is independent of the T1 used in the EPG-based dictionary (matched T2s were the same for the three generated signals and equal to 52 ms). Therefore, for the phantom and in vivo experiments, we kept the T1 constant at 1100 ms.

**
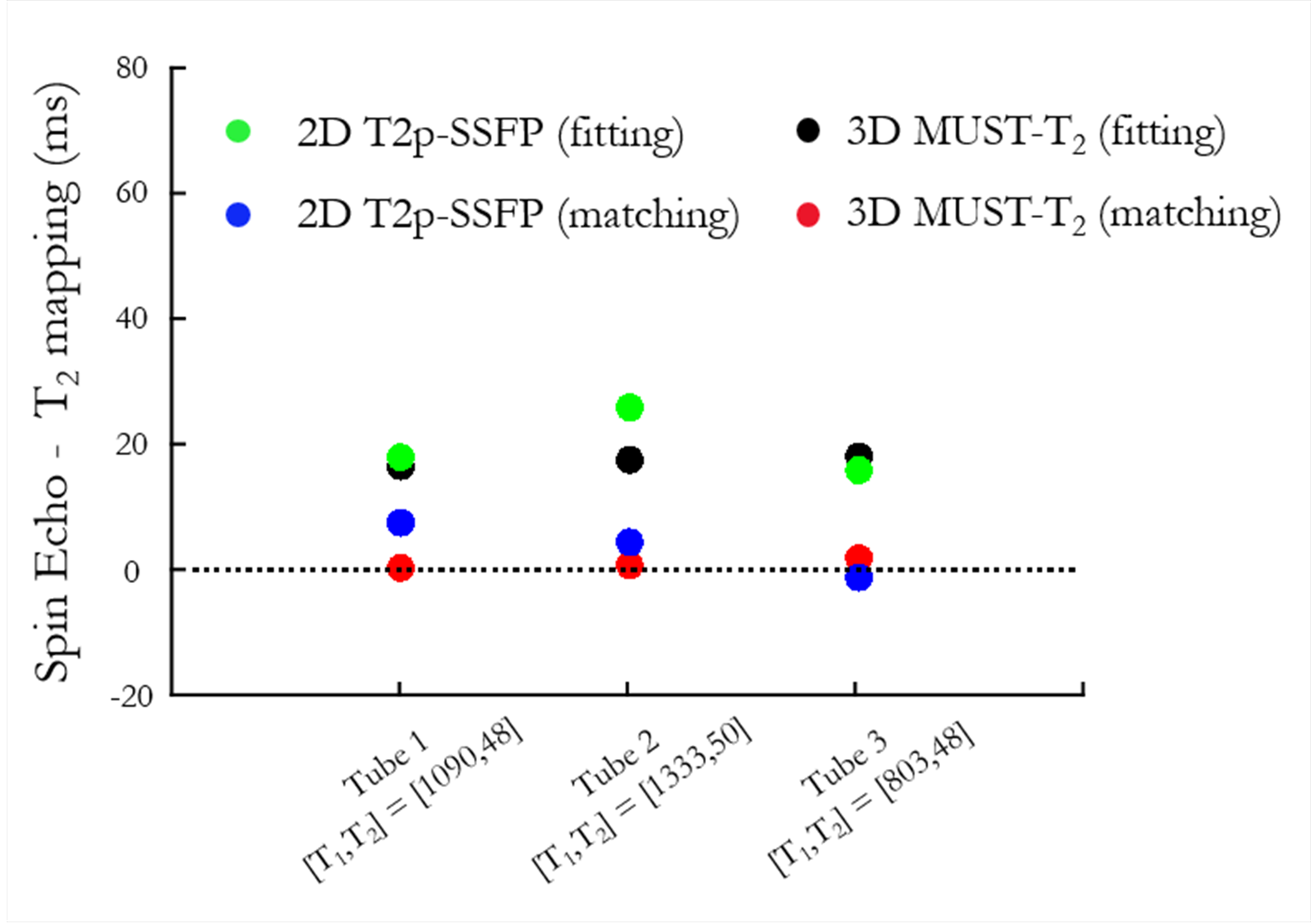
**

**Supporting Information Figure S4** Conventional mono-exponential fitting and dictionary-based matching for 2D T2p-SSFP T2 mapping in comparison to the proposed 3D MUST-T2 sequence for the phantom study. The proposed 3D acquisition with mono-exponential fitting is also included for comparison purposes. Accurate phantom T2 values, in agreement with reference spin echo, were obtained with the proposed 3D MUST-T2 sequence with dictionary-based matching, however bias is observed with the proposed acquisition when mono-exponential fitting is employed. Bias is also observed with the conventional (linear phase encoding) 2D T2p-SSFP mapping with mono-exponential fitting, however this bias is significantly reduced when dictionary-based matching is employed.

**
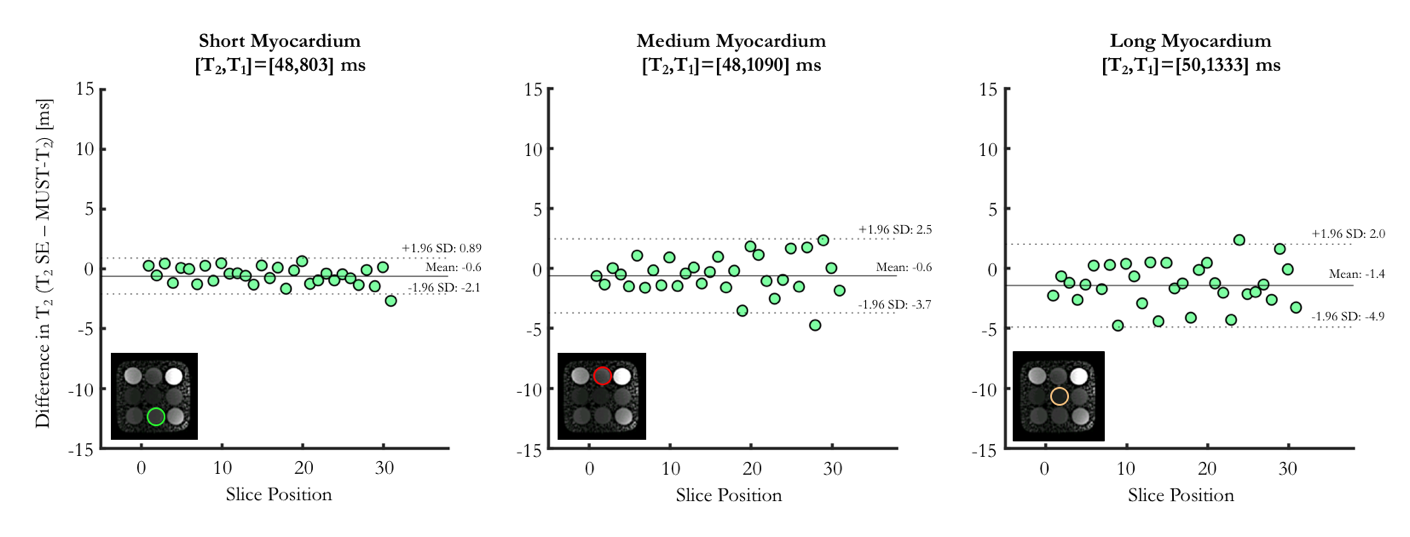
**

**Supporting Information Figure S5** Spatial T2 uniformity over the slice direction is assessed for the phantom study for three vials (corresponding to short, medium and long T1 myocardium). The solid line is the average difference between gold-standard spin echo and the proposed 3D MUST-T2 mapping sequence, and the dashed lines represent the mean ± two standard deviations between the two techniques. Good T2 uniformity can be observed with the proposed technique. The mean difference in T2 for the vial corresponding to short T1 myocardium was -0.6 ms [±95% confidence interval (CI) = -2.1/0.89 ms), -0.6 ms [±95% CI = -3.7/2.5 ms) for the medium T1 myocardium, and -1.4 ms [±95% CI = -4.9/2.0 ms) for the long T1 myocardium.

**
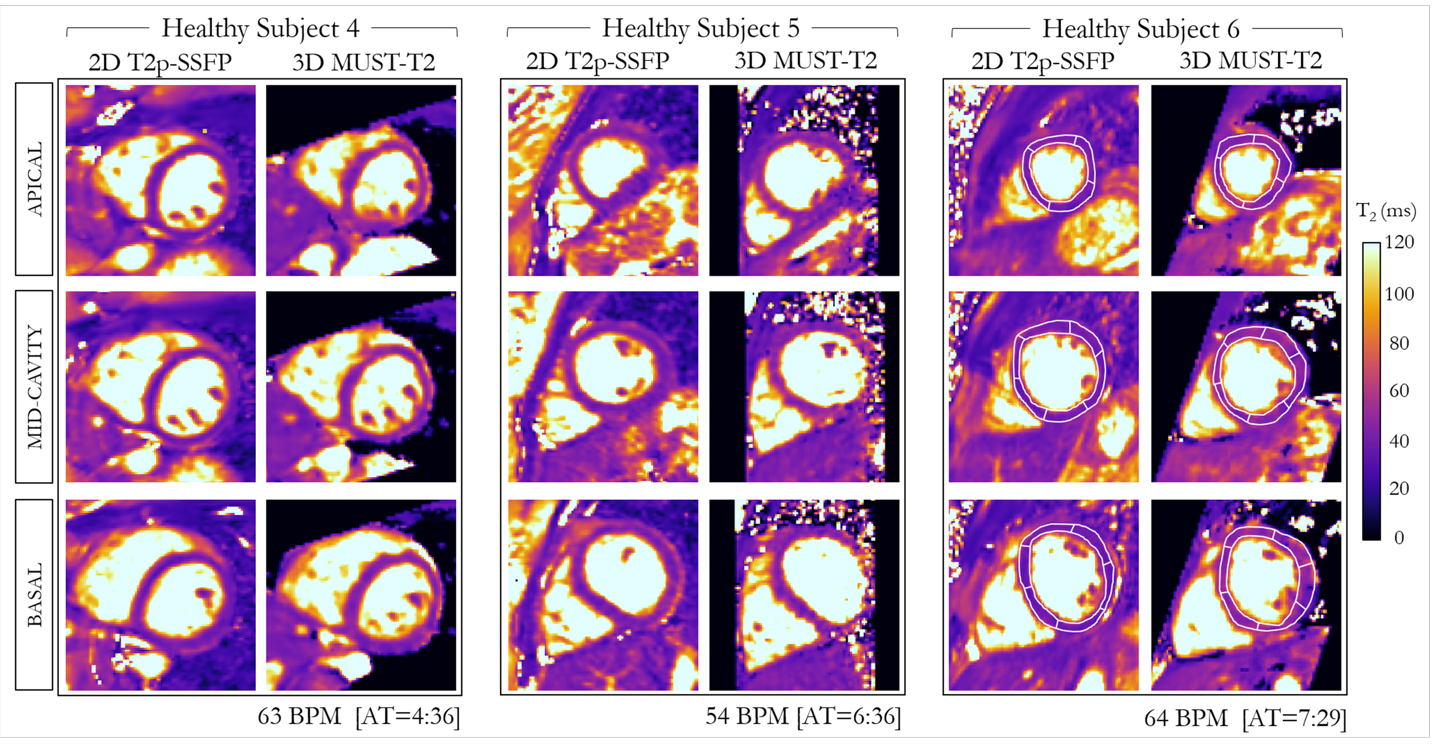
**

**Supporting Information Figure S6** T2 maps obtained using the proposed free-breathing 3D MUST-T2 mapping sequence and the conventional breath-held 2D T2p-SSFP sequence are shown for three additional healthy subjects. 3D MUST-T2 slices were reformatted to short-axis to match the 2D T2 map acquisitions. Representative 16 AHA segments are shown to illustrate how much spatial information was considered for T2 calculation. Acquisition times are expressed as [min:sec]. Abbreviations: BPM, beats per minute; AT, acquisition time.

**
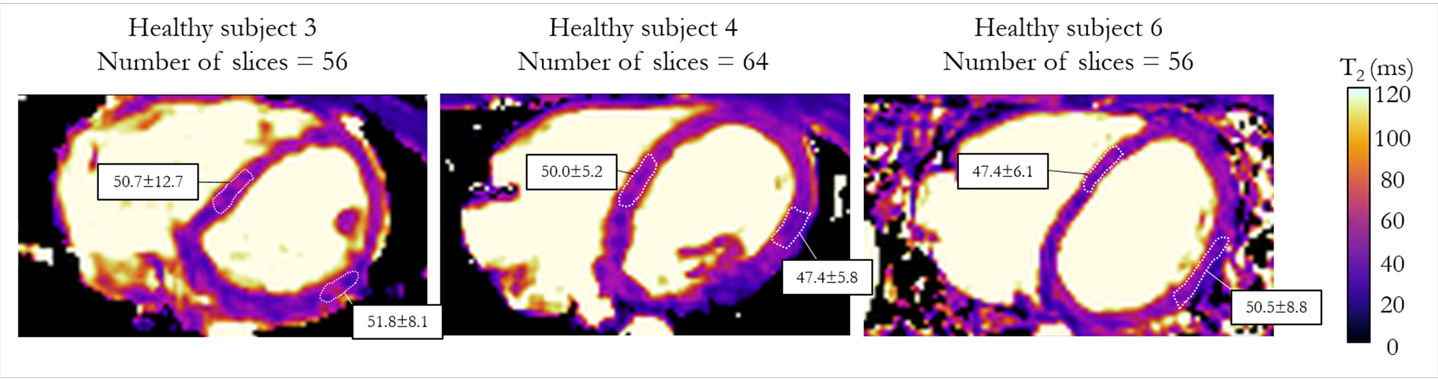
**

**Supporting Information Figure S7** Axial view of T2 maps acquired using the proposed 3D T2 mapping sequence on 3 healthy subjects. Number of slices was adjusted per subject to cover the left ventricle in the anterior-posterior direction.

**
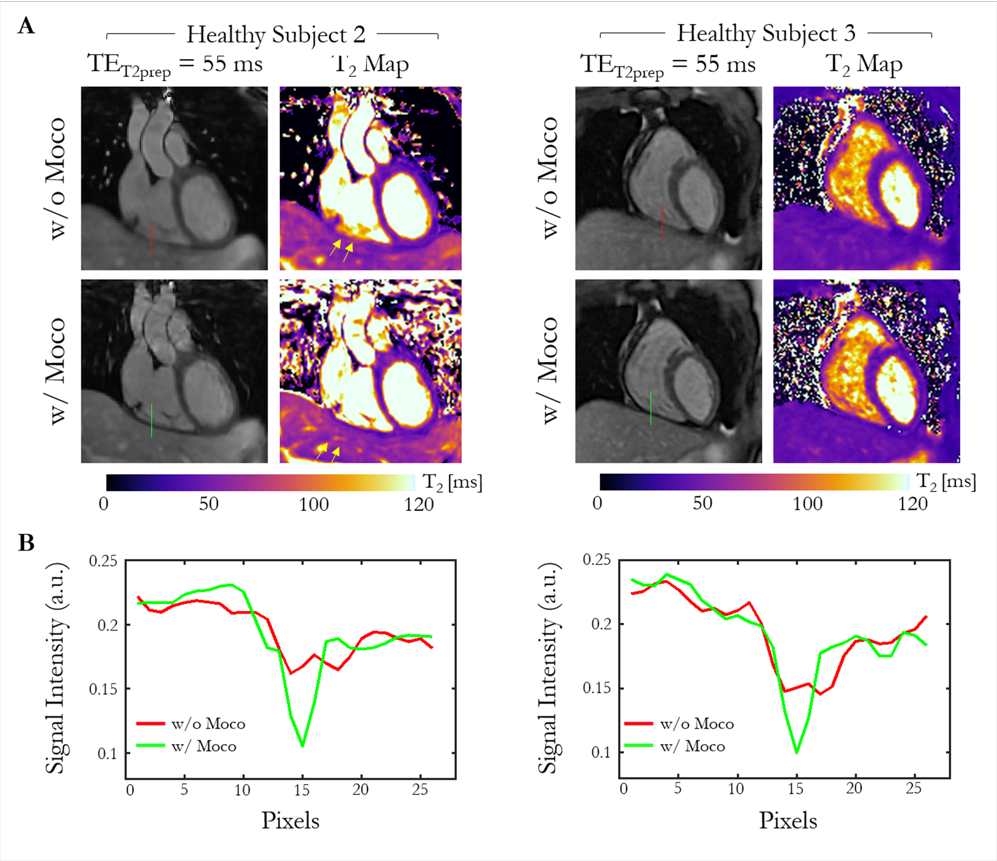
**

**Supporting Information Figure S8** Impact of iNAV-based beat-to-beat translation motion correction on 3D MUST-T2 map is shown for two healthy subjects. Reconstructed T2w images (TE_T2prep_ = 55 ms) are shown before and after motion correction with the corresponding T2 maps (A). Better visualization of the myocardium can be observed after motion correction with clear delineation of cardiac structures and myocardial walls. Note the blurring observed on the non-motion corrected T2 maps. Plots showing the intensity profiles, taken on the T2w images through the heart-liver interface, are shown in (B).

**
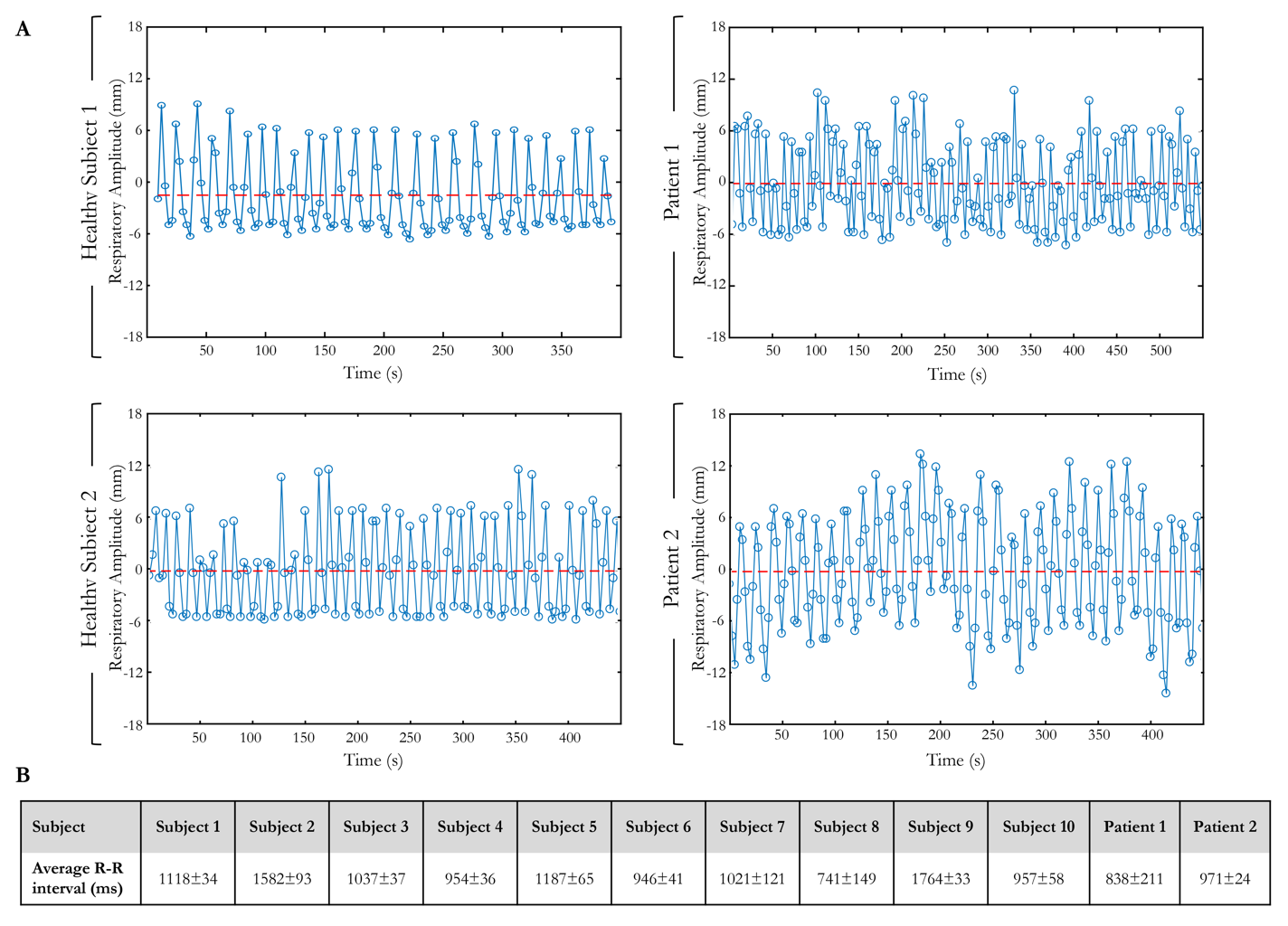
**

**Supporting Information Figure S9** (A) Foot-head respiratory displacements of the heart obtained from the 2D image navigators at each heartbeat are shown for 2 representative healthy subjects (left) and 2 patients (right). The end-expiration position is used as reference for translational motion estimation. While regular breathing patterns can be observed on the healthy subjects, more irregular breathing patterns with strong motion amplitudes are observed on patient 1 and patient 2. (B) Average R-R intervals are shown for each healthy subject and patient. Patient 1 presented with irregular cardiac rhythm (R-R = 838±211 ms).
